# Force-induced melting and S-DNA pathways for DNA overstretching exhibit distinct kinetics

**DOI:** 10.1093/nar/gkae1183

**Published:** 2024-12-09

**Authors:** Vinoth Sundar Rajan, Sune Levin, Micah J McCauley, Mark C Williams, Ioulia Rouzina, L Marcus Wilhelmsson, Fredrik Westerlund

**Affiliations:** Department of Life Sciences, Chalmers University of Technology, 412 96 Gothenburg, Sweden; Department of Chemistry and Chemical Engineering, Chalmers University of Technology, 412 96 Gothenburg, Sweden; Department of Life Sciences, Chalmers University of Technology, 412 96 Gothenburg, Sweden; Department of Physics, Northeastern University, Boston, MA 02115,, USA; Department of Physics, Northeastern University, Boston, MA 02115,, USA; Department of Chemistry and Biochemistry, The Ohio State University, Columbus, OH 43210, USA; Department of Chemistry and Chemical Engineering, Chalmers University of Technology, 412 96 Gothenburg, Sweden; Department of Life Sciences, Chalmers University of Technology, 412 96 Gothenburg, Sweden

## Abstract

It is widely appreciated that double stranded DNA (dsDNA) is subjected to strong and dynamic mechanical forces in cells. Under increasing tension B-DNA, the most stable double-stranded (ds) form of DNA, undergoes cooperative elongation into a mixture of S-DNA and single stranded DNA (ssDNA). Despite significant effort, the structure, energetics, kinetics and the biological role of S-DNA remains obscure. We here stretch 60 base pair (bp) dsDNA oligonucleotides with a variable number of tricyclic cytosine, tC, modifications using optical tweezers. We observe multiple fast cooperative and reversible two-state transitions between B-DNA and S-DNA. Notably, tC modifications increase the transition force, while reducing the transition extension and free energy due to progressively increasing fraying of the dsDNA ends. We quantify the average number of bps undergoing the B-to-S transition, as well as the free energies and rates. This allows us to reconstruct the B-to-S free energy profiles in absence of force. We conclude that S-DNA is an entirely force-induced state, and that the B-to-S transition is much faster than internal dsDNA melting. We hypothesize that S-DNA may have a role as a transient intermediate in, for example, molecular motor-induced local dsDNA strand separation.

## Graphical Abstract

**Graphical Abstract:**
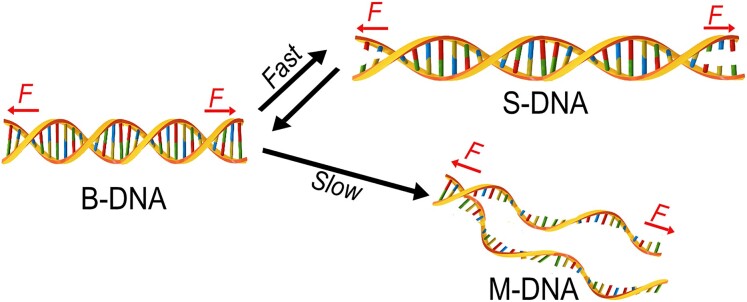

## Introduction

Double stranded (ds) DNA can be extended, twisted and unwound during its repair, expression or other genome maintenance processes (1–3). Single-molecule manipulation techniques allow studies of the mechanical behavior of single DNA molecules by applying external force and/or torque. DNA mechanics has been investigated using different techniques including optical (4,5) and magnetic (6,7) tweezers, as well as atomic force microscopy (8,9). Such experiments apply and measure forces in the pico- to micro-Newton (N) range with nanometer (nm) resolution. Smith *et al.* (4) and Cluzel *et al.* (10) reported in the 1990s that stretching the common double stranded B-form of λ-DNA (∼48 500 base pairs (bps)) along its helix axis leads to a highly cooperative and well-defined elongation by 70%, without strand separation, at forces of ∼60–70 pN, followed by a steep force increase of the new dsDNA structure that is ∼3-fold less extensible than B-DNA. This overstretched form of dsDNA was termed S-DNA. Theoretical models and molecular dynamics simulations provided molecular details of the B-to-S transition (11–15). S-DNA is a base-paired and largely unwound ladder-like structure (13) with distorted, inclined base pairing (10,16), leading to an increased distance between adjacent bases (0.58 nm) (4) and 33–37 bps per helical turn (14) as compared to 0.34 nm and 10.5 bps for B-DNA. However, modeling did not offer a unique S-DNA structure and extension, which instead appeared dependent on the geometry of the force application (5′ versus 3′ of the same or opposite strand), the DNA sequence and the rate of stretching, yielding free energies of B-DNA overstretching into S-DNA that were several-fold larger than their experimental values (10–12,14,17), most likely due to the limited computational stretching time of ns-ms, while this process is occurring on a seconds time scale in experiments.

It has been difficult to experimentally distinguish S-DNA from melted DNA (M-DNA) (17–19) that can also be formed during overstretching, as the elongation, the force and the free energy of S-DNA and M-DNA are quite similar. Melting of B-DNA during the overstretching transition is strongly supported by experiments showing that most conditions that alter thermal B-DNA melting also alter the overstretching force in the same way (20–22). Several studies have demonstrated a dependence of the overstretching force on pH (20), ionic strength (21), temperature (22) and the presence of hysteresis when reannealing to B-form (4). Force-induced melting has been further supported in experiments using DNA-binding molecules like glyoxal (23) and combinations of force spectroscopy with fluorescence microscopy (24,25). The major distinction between these two modes of dsDNA overstretching has been shown to lie in their temperature dependence. While dsDNA melting is always strongly promoted by higher temperatures, the B-to-S transition is almost insensitive to the temperature and seems to be even slightly stabilized by its increase (26).

While the sequence dependence of B-DNA melting has been extensively studied (27,28), systematic analysis of the sequence dependence of the B-to-S transition is lacking. This is because the same conditions that promote the B-to-S transition in GC-rich DNA oligomers will lead to force-induced melting in AT-rich DNA (29–32). Bosaeus *et al.* demonstrated that, based on the sequence, a 60-bp oligonucleotide can undergo either a reversible B-to-S transition or force-induced melting (fraying where DNA melts from free ends and internal melting where base pairs are separated in some regions and intact in others) (24,31,32). Another important distinction between the B-to-S and B-to-M transitions seems to be the kinetics. The kinetics of the thermal melting of both polymeric and oligomeric dsDNA has been studied extensively and is known to be relatively slow, especially in low salt (27). Force-induced fraying of polymeric dsDNA results in slow re-annealing of the strands, leading to profound stretch-release hysteresis (29,33). So far only two studies have addressed the B-to-S transition kinetics in λ-DNA using optical tweezers (34,35).

**Figure 1.**
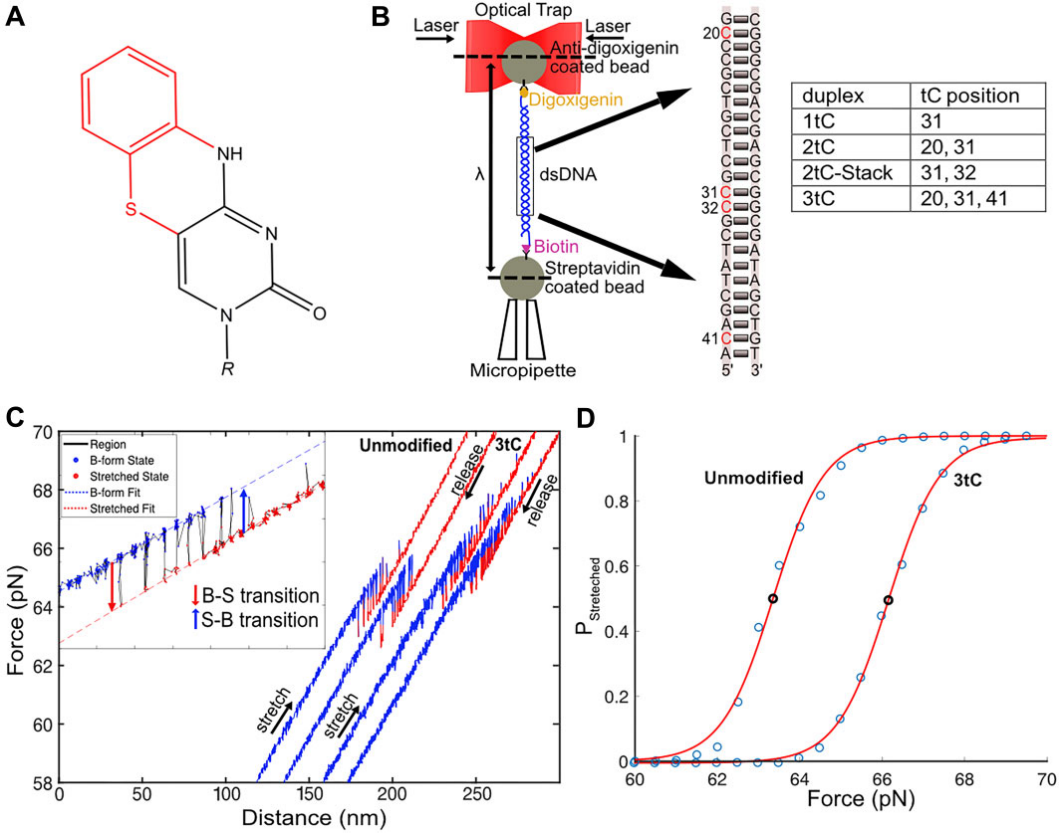
Experimental setup. (**A**) Structure of the tC nucleobase; highlighted in red is the additional aromatic ring structure absent in natural cytosine. (**B**) Schematic representation of the optical tweezers setup. Inset shows the tC modifications in the central 30 bp region at different positions (red). The table shows the specific modifications for each tC-containing oligonucleotide. (**C**) FDCs for Unmodified and 3tC oligonucleotides showing both B-to-S transitions and S-to-B transitions with B-DNA in blue and S-DNA in red during stretch and release. The stretch and release parts of each cycle, as well as Unmodified versus 3tC curves, are shifted relative to each other along the Distance axis for better visualization. Inset: Zoom of a curve illustrating the B- (blue) and S- (red) states and their linear fits (blue and red dashed lines). (**D**) The probability of finding the molecule in the S-state as a function of force is fitted with a two-state model (see Equation S1) to obtain the transition force, *F_tr_* (shown in the plot as black circles).

Modified nucleobases provide a way to alter the base stacking and/or hydrogen bonding interactions with minimal perturbation to the native structure of nucleic acids (36–38). Recently, single-molecule techniques have been used to study nucleic acids with base modifications, base analogues and mismatches, to understand how these chemical modifications change the mechanical properties and stability of nucleic acids (39–43). 2,6 diaminopurine (DAP), a nucleobase analogue of adenine, has been used to manipulate the mechanical properties of dsDNA via an additional third hydrogen bond with thymine. The DAP substitution resulted in a DNA overstretching transition switching from melting to B-to-S transition as a result of its differential effect of DAP stabilizing B-DNA with respect to melting, and destabilizing with respect to its B-to-S transition (44,45), providing a convincing example that both types of DNA overstretching transitions depend on DNA sequence and in a different way. The fluorescent tricyclic cytosine analogue tC (Figure 1A) base pairs with guanine using three hydrogen bonds and slightly increases base stacking due to the extended aromatic rings without distorting the DNA backbone conformation (46–49). This increased stacking by tC in general leads to an increase in the DNA melting temperature as well as to an increased unzipping force of a tC-modified DNA hairpin, reporting on the B-DNA stabilization with respect to strand separation, by ∼1.3 k_B_T per each tC modification (50).

The current study characterizes the kinetics and thermodynamics of the B-to-S transition in a 60 bps, 60% GC DNA duplex modified with one, two or three tC bases. We also study the effect of base rearrangements that minimize stacking interactions, while maintaining the same 60% GC composition. We find that each tC modification progressively stabilizes the B-form duplex relative to S-DNA, just as it stabilizes B-DNA with respect to melting. This effect, however, is masked by an increasing B-to-S transition force leading to more bps fraying from the duplex ends before the B-to-S transition occurs. This, in turn, leads to fewer bps undergoing the B-to-S transition, shorter transition extension, and lower net transition free energy. These observations allow us to characterize the free energy per bp, elongation and force for the B-to-S transition. Quantifying the number of bps undergoing B-to-S transition allows us to analyze the free energy and kinetics of the B-to-S transition as a function of the dsDNA oligomer length. We find that the B-to-S transition is fast (∼1–10 s^−1^) and its rate depends only weakly on the dsDNA stability, solution ionic strength and oligonucleotide length. In contrast, the dsDNA oligonucleotide melting transition is known to become very slow in low salt, at higher temperatures and when the local ssDNA strand concentration is low. Our results suggest a possible novel physiological role of S-DNA as a force-induced transient intermediate on the pathway to internal DNA melting, or a possible substrate for the recognition by yet unknown proteins.

## Materials and methods

### Design of DNA duplexes for overstretching studies

The DNA constructs used in this work consist of a 60-bp double-stranded region and single-stranded handles on both ends for the attachment of beads. The duplex DNA is obtained from the hybridization of complementary oligonucleotides (oligos 1 and oligos 2). The single stranded 60-mer oligonucleotides for unmodified (oligo 1–1) and with tCs (purchased from Glen Research, Sterling VA, USA) incorporated at various positions and number (oligo 1–2, oligo 1–3, oligo 1–4, oligo 1–5) as well as the complementary oligonucleotide (oligo 2–1) were synthesized and supplied by ATDBio, Southampton, UK. The oligos were hybridized in-house to form DNA duplexes: Unmodified, 1tC, 2tC, 2tC-Stack and 3tC with 60% GC content (Figure 1B and Supplementary Table S1). Likewise, 60-mer oligonucleotides (oligo 1–6, oligo 1–7) and their complementary oligonucleotide (oligo 2–2, oligo 2–3) with some or all subsequent purine bases in the central 30-bp region substituted by pyrimidines, were synthesized, supplied and hybridized in-house as Unmodified-Int and Unmodified-Low conserving 60% GC content (Table S1). The handles were formed by extension of the 3′-ends using Terminal Transferase (Merck, Ref. #: 3 333 566 001) at 37°C. The 3′-ends of oligos 1 were tailed using dUTP-digoxigenin (Digoxigenin-11-dUTP, Roche, Ref. #: 11 558 706 910) to dATP (Sigma-Aldrich, Ref. #: 11 140 965 001) in a ratio of 1:10 while the 3′-ends of oligos 2 were tailed using dUTP-Biotin (Biotin-16-dUTP, Sigma-Aldrich, Ref. #: 1 109 307 910) to dATP in the same ratio. The tailed oligos 1 and oligos 2 were purified using the Qiaquick Nucleotide Purification Kit (Qiagen, Ref. #: 74 104). The different duplex DNAs were obtained by mixing oligos 1 and oligos 2 and hybridizing them in a PCR instrument with the following steps: heating at 95°C for 1.0 min, cooling from 95 to 80°C in 10 s, heating at 80°C for 10 s, cooling from 80 to 40°C at 0.5°C/10 s and from 40 to 10°C at 0.5°C/20 s. The DNA constructs obtained were aliquoted and stored at −20°C and used for the experiments. The single-stranded tails at the 3′-ends of the dsDNA constructs with incorporated digoxigenin or biotin-modified nucleotides allowed the attachment of DNA constructs to polystyrene beads coated with digoxigenin antibodies or streptavidin (see Supplementary Section 1 for details), respectively.

### Optical tweezers experiments

Single-molecule experiments were performed using optical tweezers (see Supplementary Section 2 for more details on setup) by attaching the 3′-end of oligos 1 to anti-digoxigenin-coated beads and the 3′-end of oligos 2 to streptavidin-coated beads. The streptavidin-coated bead was held on the micropipette and the anti-digoxigenin bead was trapped in the optical trap, as shown in Figure 1B. A tether containing a single dsDNA oligonucleotide was established between the two beads with each end of the dsDNA oligonucleotide attached to the bead by only one strand. The dsDNA oligonucleotide force-distance curves (FDCs) were obtained by varying the force between minimum (F_min_) and maximum (F_max_) values below and above (which no B-to-S transitions ever occurred) and recording their corresponding distance (λ) between center of trap and the center of bead in the micropipette. The trap, with a stiffness of 0.1 pN/nm, was moving at a constant velocity (50 nm/s) from F_min_ to F_max_, such that the net stretch-release cycle took ∼4 s. Over that time the dsDNA oligonucleotide underwent multiple B-to-S and S-to-B transitions (bistability), observed as sudden jumps in force at a given extension between the B-DNA and S-DNA stretching curves (Figure 1C). The relative stiffness of the molecular construct compared to the stiffness of the optical trap, K_trap_, in this force region makes the effective stiffness K_eff_ ∼ K_trap_, (i.e the contribution from the handles and the experimental system is negligible) and the force is thus varied with an approximately constant loading rate. Data were recorded at a frequency of 1 kHz. All the experiments were carried out at a constant temperature of 23 ± 1°C. Measurements were performed in a buffer containing 10 mM Tris pH 7.4, 1 mM EDTA, 1 M NaCl, unless indicated.

## Results

### Tricyclic cytosine (tC) incorporation stabilizes B-DNA with respect to S-DNA

**Figure 2.**
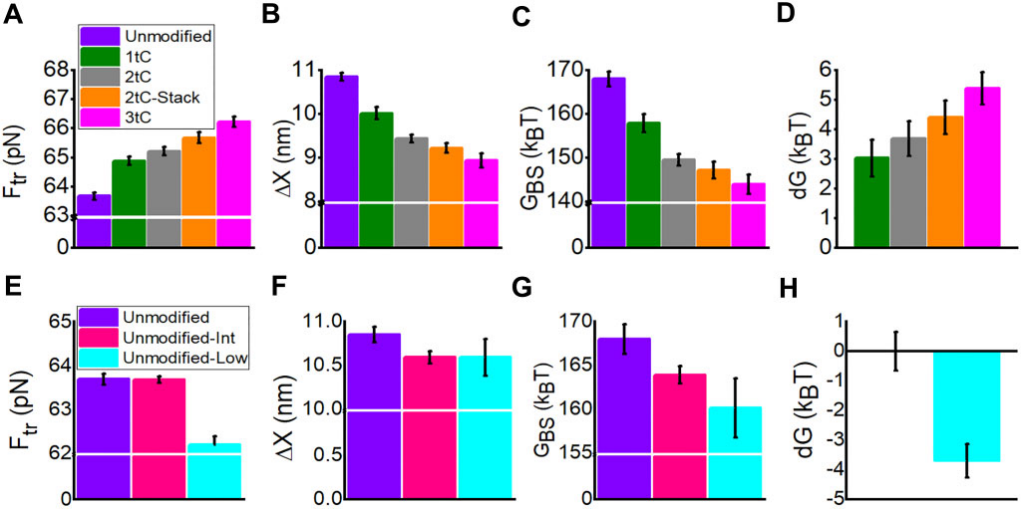
Equilibrium properties of the B-to-S transitions for dsDNA oligonucleotides with tC incorporations (A-D) and varying number of purine pairs (E-H). (**A**, **E**) Changes in transition force, ${{F}_{tr}}$; (**B**, **F**) average extension, $\Delta X$; and (**C**, **G**) free energy, ${{G}_{BS}}$, obtained from measurements. (**D**, **H**) Calculated change in stability of dsDNA $dG$ due to modifications. All data obtained at room temperature (23 ± 1°C) and 1 M NaCl. Unmodified (violet), 1tC (green), 2tC (grey), 2tC-Stack (orange) and 3tC (magenta). Unmodified (violet), Unmodified-Int (pink) and Unmodified-Low (cyan).

Mechanical stretch-release cycles of the dsDNA oligomers were performed at a constant velocity (50 nm/s) from 55 to 72 pN such that each cycle was completed within ∼4 s (unless otherwise noted, see ‘Materials and methods’ for details). Typical FDCs during stretch and release for the Unmodified and 3tC oligonucleotides are shown in Figure 1C (Supplementary Figure S1A-F for the other oligonucleotides). All constructs exhibit multiple cooperative two-state transitions within a narrow force interval. Each transition is observed as an abrupt force drop or increase as the dsDNA oligonucleotide undergoes a transition from the B-form to the overstretched S-form (B-to-S) and back (S-to-B), respectively. As the two strands are not crosslinked to each other, the observed two-state reversible transitions clearly do not involve complete strand separation, but are rather transitions between two double-stranded states of the DNA duplex. The FDCs contain extensive information on both equilibrium and kinetic properties of the B-to-S transition. We first analyzed these curves to obtain the equilibrium transition force ${{F}_{tr}}$ (the force at which the probability of being in the S and B states are the same), B-to-S transition extension $\Delta X$, and the equilibrium transition free energy ${{G}_{BS}}$, as described in Supplementary Section 3 and 4, shown in Figure 2 and reported in Table 1. The B-to-S transition extension is considered only during the abrupt stepwise transition, and the trap and the handles are stiff enough so that they do not contribute to the changes in the extension during this transition, within the uncertainty of these results. Thus, this length change may be used to calculate the free energy associated with the B-to-S transition only.

**Table 1.** Equilibrium properties of the B-to-S transition in the wild-type, base-modified and rearranged dsDNA duplex

dsDNA	m^a^	${{F}_{tr}}$ ^b^ (pN)	${\mathrm{\Delta }}X$ ^b^ (nm)	${{G}_{BS}}$ ^b^ (k_B_T)	$n$ ^c^ (bps)	$g$ ^c^ (k_B_T/bp)	$dG$ ^d^ (k_B_T)
Unmodified	176	63.7 ± 0.1	10.8 ± 0.1	168.0 ± 1.7	47.2 ± 0.4	3.56 ± 0.01	0
1tC	117	64.9 ± 0.1	10.0 ± 0.1	157.9 ± 2.1	43.5 ± 0.6	3.63 ± 0.01	3.04 ± 0.62
2tC	82	65.2 ± 0.1	9.4 ± 0.1	149.5 ± 1.3	41.0 ± 0.4	3.65 ± 0.01	3.69 ± 0.58
2tC-Stack	81	65.7 ± 0.2	9.2 ± 0.1	147.2 ± 1.9	40.1 ± 0.5	3.67 ± 0.01	4.41 ± 0.57
3tC	118	66.2 ± 0.2	8.9 ± 0.1	142.7 ± 2.0	38.5 ± 0.6	3.70 ± 0.01	5.39 ± 0.55
Unmodified-Int	237	63.7 ± 0.1	10.6 ± 0.1	163.9 ± 1.0	46.0 ± 0.3	3.56 ± 0.01	−0.01 ± 0.65
Unmodified-Low	241	62.2 ± 0.2	10.6 ± 0.2	160.1 ± 3.4	46.1 ± 0.9	3.48 ± 0.01	−3.69 ± 0.66
Unmodified-200 nm/s	667	62.9 ± 0.2	10.5 ± 0.3	160.1 ± 4.3	45.5 ± 1.3	3.52 ± 0.01	0
3tC-200 nm/s	124	66.1 ± 0.1	8.4 ± 0.1	134.4 ± 1.1	36.4 ± 0.3	3.69 ± 0.01	6.19 ± 0.52
Unmodified-150 mM Na^+^	55	62.7 ± 0.1	10.2 ± 0.1	154.8 ± 1.1	44.2 ± 0.3	3.50 ± 0.01	0

^a^Number of stretch and release cycles (m) obtained for 5–10 different bead pairs.

^b^Experimental parameters from the curves.

^c^Number of bps estimated using $n = {\mathrm{\Delta }}X/{{x}_{BS}}$, where ${{x}_{BS}} = 0.23\frac{{nm}}{{bp}}$ is the per bp DNA extension during the B-to-S transition known from polymeric dsDNA overstretching studies (35,51), and transition free energy per bp using $g = {{G}_{BS}}/n$.

^d^Net duplex stabilization $dG$ due to modifications was calculated using Equation S6. The reported values are the mean ± standard error of mean (SEM). Measured in 10 mM Tris pH 7.4, 1 mM EDTA, 1 M NaCl, at 23 ± 1°C and a pulling rate of 50 nm/s unless indicated. Unmodified and 3tC duplexes were also measured at 1 M and 200 nm/s pulling rate and 150 mM NaCl and 50 nm/s pulling rate. The data for 3tC at 150 mM NaCl is shown in Supplementary Table S6. Note: The upper-case values, including length and free energies, are for the *n-*bps DNA duplex while all lower-case values, are per bp. Also, G with subscripts denotes an equilibrium free energy.

Each additional tC modification induces a progressive upward shift in the range of transition forces with the most probable force, ${{F}_{tr}}$ (determined as described in Supplementary Section 3Equation S1 and in Figure 1D), increasing from 63.7 ± 0.1 pN (Unmodified) to 66.2 ± 0.2 pN (3tC), consistent with the expected duplex stabilizing effect of tC (50) (Table 1 and Figure 2A). Contrastingly, the B-to-S transition extension ($\Delta X$) decreases with the number of tC incorporations, from 10.8 ± 0.1 nm for Unmodified to 8.9 ± 0.2 nm for 3tC (Table 1 and Figure 2B). The equilibrium B-to-S transition free energy (${{G}_{BS}}$), proportional to the product of ${{F}_{tr}}$ and $\Delta X$, also decreases significantly with increasing number of tCs in the duplex, from 168.0 ± 1.7 k_B_T for Unmodified to 142.7 ± 2.0 k_B_T for 3tC (Figure 2C). In other words, introducing three tC modifications decreases the free energy of the B-to-S transition by ∼25 k_B_T. We obtained very similar (slightly lower) values of ${{G}_{BS}}$ using the Bennett's acceptance ratio method (Supplementary Section 4, Supplementary Figure S2 and Supplementary Table S2) which provides a firm basis for the subsequent analysis and conclusions. Does this mean that the tC modifications destabilize B-DNA relative to S-DNA, in contrast to their opposite effect on B-DNA melting? A closer look at the data shows that the profound decrease in ${{G}_{BS}}$ comes largely from the decrease in$\ \Delta X$ for the tC-modified duplexes. This decrease in $\Delta X$ would not be expected if all 60 bps of the B-DNA oligomer was undergoing the B-to-S transition. It has been established that at high salt concentrations and at room temperature (as in our experiments), polymeric dsDNA overstretches into the S-state at ∼65 ± 3 pN with a 1.7-fold length extension, corresponding to an elongation per bp of ${{x}_{BS}} = 0.23\ nm/bp$ (35,51). Therefore, the expected elongation for the 60 bp dsDNA during its complete cooperative B-to-S-transition is ∼13.8 nm, significantly larger than the ∼9–11 nm measured in our experiments. A similar ∼10 nm elongation during the B-to-S transition for 60 bp DNA duplexes was observed by Bosaeus *et al.*, (31) and was then interpreted as overstretching into a ∼1.5-fold longer dsDNA state. Another possible explanation for the shorter elongation is that not all bps undergo the B-to-S transition. Using this assumption, the known per bp elongation ${{x}_{BS}}$ for polymeric dsDNA and the measured average duplex extension $\Delta X$ during the transition (Supplementary Section 5 and Equation S4) can be used to calculate the average number of bps undergoing the B-to-S transition, $n$ (Table 1).

This analysis suggests that for the Unmodified oligonucleotide, only ∼47 bps undergo the B-to-S transition and that this number decreases gradually to 38 bps for 3tC (Table 1). We therefore propose that the B-to-S transition occurs not in the whole 60 bp duplex, but rather in the part of the duplex that remains in the B-DNA form at the transition force. The rest of the bps presumably fray from the end(s) prior to the B-to-S transition in the rest of the duplex. Duplex DNA fraying at forces well below the B-to-S transition has been observed previously (23,24,40,52). The fact that nine fewer bps undergo the B-to-S transition in the 3tC DNA molecule explains the ∼25 k_B_T decrease in equilibrium transition free energy (Figure 2C and Table 1). Importantly, the transition free energy per bp $(g$) calculated as $g$*=G_BS_/n* (Equation S5) appears to be almost constant for all duplexes, varying by $ \le 3\%$ (3.56 and 3.70 k_B_T/bp, respectively. Here and below all free energies are in the units of 1 k_B_T = 0.59 kcal/mol at physiological temperature) between Unmodified and 3tC (Table 1), supporting the hypothesis that the free energy of the B-to-S transition in dsDNA oligonucleotides is proportional to the number of bps.

We can also calculate the increase in the B-to-S transition free energy due to each tC modification by comparing the measured transition free energy in the modified oligonucleotide and the free energy of the unmodified oligonucleotide of the same length (Equation S6 and Table 1). On average, each tC modification stabilizes the duplex by ∼2 k_B_T. This is a slightly stronger stabilizing effect on the B-to-S transition compared to the previously measured effect of tC modification on force-induced unzipping, or melting, which was ∼1.3 k_B_T per bp (50). Duplex stabilization due to two adjacent tC modifications in the 2tC-Stack duplex shows approximately the same duplex stabilization as two independently positioned tC modifications in the 2tC duplex, suggesting no additional synergistic effect of tC stacking on the B-to-S transition.

### Minimizing the number of adjacent purines slightly favors the B-to-S transition while stabilizing B-DNA against melting

Since we observed a significant stabilizing effect of tC on the equilibrium B-to-S transition free energy, we next explored the effect of stacking for the four natural bases on the B-to-S transition. Purines have larger aromatic systems than pyrimidines and are hence expected to stack more when positioned adjacently in the same strand (46–48). We therefore substituted all or a fraction of subsequent purines in the central 30 bp part of the DNA while keeping the GC content of the duplex at 60%, (Unmodified-Low and Unmodified-Int, respectively, Supplementary Table S1). Unmodified-Low (with no adjacent purines) showed a significantly lower ${{F}_{tr}}$ (62.2 ± 0.2 pN), suggesting duplex destabilization compared to Unmodified-Int (63.7 ± 0.1 pN) and Unmodified (63.7 ± 0.1 pN) (Table 1 and Figure 2E). Moreover, $\Delta X$ decreased slightly for both duplex variants (Table 1 and Figure 2F). The B-to-S transition free energy (${{G}_{BS}}$) also decreased from 168 ± 1.7 k_B_T (Unmodified) to 163.9 ± 1.7 k_B_T (Unmodified-Int) and 160.1 ± 3.4 k_B_T (Unmodified-Low) (Figure 2G). While these effects are consistent with our expectations of weaker stacking making the B-to-S transition easier, the effect is quite modest. It is also opposite to the effect of the same base substitutions on the free energies of melting for Unmodified, Unmodified-Int and Unmodified-Low, predicted by the DINAMelt program (53) to increase by 4 k_B_T and 7 k_B_T, respectively (Supplementary Table S3). This is an interesting result, showing another example (in addition to DAP substitutions (44,45)) that the B-to-S and B-to-M transitions can be affected in opposite ways by the same DNA sequence variations.

### The transition state lies midway between the B- and S-states

**Figure 3.**
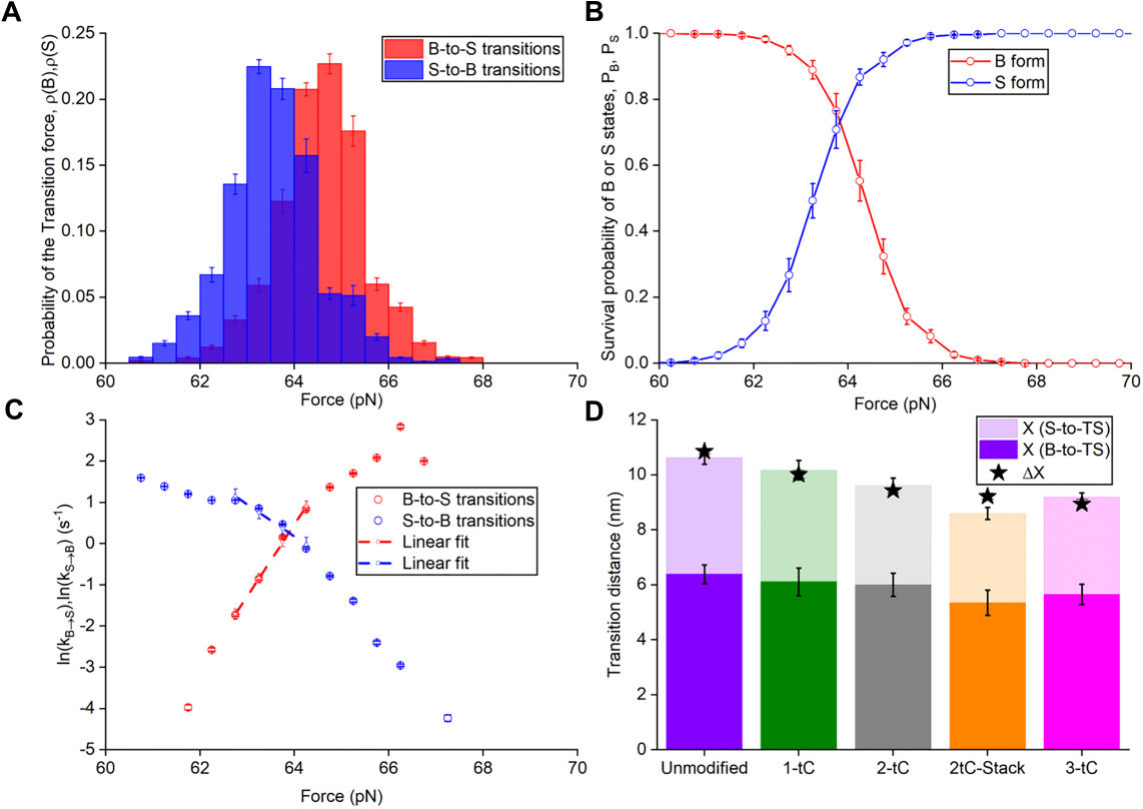
Finding the duplex extension at the B-to-S transition free energy barrier using the Bell-Evans (BE) model. Probability distribution of transition forces of the B-to-S (red) or S-to-B (blue) transition (**A**), survival probability (**B**) and the transition rates versus force for the Unmodified DNA duplex (**C**). Dashed lines are the ${\mathrm{ln}}({{k}_{B \to S}})$ and ${\mathrm{ln}}({{k}_{S \to B}})$ fits to the BE model (Equation S9) in the vicinity of the transition force ${{F}_{tr}}$. (**D**) Fitted extensions $({{X}_{B \to TS}}$) (filled) and $( {{{X}_{S \to TS}}} )$ (transparent) from the B- and S- states, respectively, to the transition state (TS) for the duplexes. Also presented is the sum ${{X}_{BS}} = {{X}_{B \to TS}} + {{X}_{S \to TS}}$ (net height of each bar) and the directly measured transition elongation $\Delta X$ (from Figure 2B), indicated with a star for each DNA duplex.

It has previously been challenging to separate the kinetics of the B-to-S and the B-to-M transitions that often co-exist in the same DNA molecule (26,35). In our experiments we observed a clear B-to-S transition within a region of the DNA duplexes of known average length that varies between studied dsDNA constructs. Besides the average equilibrium parameters of the B-to-S transition for each dsDNA oligonucleotide variant, our stretching curves also contain extensive information on the transition kinetics, with multiple B-to-S and S-to-B transitions occurring over each stretch - release cycle. We used these data to construct the transition force probability distribution for the B-to-S and S-to-B transitions (Figure 3A for Unmodified and Supplementary Figure S1G–L for the other oligonucleotides and Supplementary Section 6 for more details) and survival probability distributions for the B- and S-states as a function of force (Figure 3B for Unmodified), calculated according to Equation S7, which can potentially provide information on the B-to-S transition kinetics (54,55). In particular, we calculate the B-to-S and the S-to-B transition rates as a function of force, ${{k}_{B \to S}}( F )$ and ${{k}_{S \to B}}( F )$ (Figure 3C for Unmodified, Supplementary Figure S1M–R for the other oligonucleotides), as described in Equation S8. We fit the ln of the rates versus force to the exponential Bell-Evans expressions (Equation S9) (39,56,57) to obtain the extensions to the transition state (TS) from the B- and S- states and their sum, $({{X}_{B \to TS}}$, ${{X}_{S \to TS}}$, ${{X}_{BS}} = {{X}_{B \to TS}} + {{X}_{S \to TS}})$, respectively, for all dsDNA oligomers (Table 2, Figure 3D and Supplementary Figure S3A). Importantly, the total transition elongation (${{X}_{BS}}$) estimated in this way (Figure 3D and Table 2) agrees well with the direct measurement of the net average B-to-S transition elongation ($\Delta X$, Table 1).

**Table 2.** Analysis of the force dependence of the B-to-S and S-to-B transition rates with the Bell-Evans approach (Equation S9) for the wild type, base modified and base rearranged dsDNA oligonucleotides

dsDNA	${{X}_{B \to TS}}{\mathrm{\ }}$ ^a^ (nm)	${{X}_{S \to TS}}$ ^a^ (nm)	${{X}_{B{\mathrm{S}}}}$ ^a^ (nm)	$n$ ^b^ (bps)	${{x}_{B \to TS}}$ ^b^ (nm/bp)	${{x}_{S \to TS}}$ ^b^ (nm/bps)	${{X}_{B \to TS}}/X$ ^c^ (%)	N ^d^
Unmodified	6.4 ± 0.3	4.2 ± 0.2	10.6 ± 0.4	46.2 ± 1.8	0.14 ± 0.01	0.09 ± 0.01	60.1 ± 4.0	25 ± 3
1tC	6.1 ± 0.5	4.1 ± 0.3	10.2 ± 0.6	44.2 ± 2.6	0.14 ± 0.01	0.09 ± 0.01	60.0 ± 6.1	28 ± 3
2tC	6.0 ± 0.4	3.6 ± 0.3	9.6 ± 0.5	41.8 ± 2.2	0.14 ± 0.01	0.09 ± 0.01	62.3 ± 5.5	42 ± 4
2tC-Stack	5.3 ± 0.5	3.2 ± 0.2	8.6 ± 0.5	37.4 ± 2.2	0.14 ± 0.01	0.09 ± 0.01	62.2 ± 6.5	44 ± 5
3-tC	5.6 ± 0.4	3.5 ± 0.1	9.2 ± 0.4	40.0 ± 1.7	0.14 ± 0.01	0.09 ± 0.01	61.4 ± 4.8	65 ± 6
Unmodified-Int	6.5 ± 0.2	4.0 ± 0.3	10.5 ± 0.4	45.8 ± 1.6	0.14 ± 0.01	0.09 ± 0.01	61.8 ± 2.8	23 ± 4
Unmodified-Low	5.6 ± 0.2	4.2 ± 0.3	9.8 ± 0.4	42.7 ± 1.8	0.13 ± 0.01	0.10 ± 0.01	57.3 ± 3.4	31 ± 5
Unmodified-200 nm/s	6.4 ± 0.3	3.6 ± 0.3	10.0 ± 0.4	43.5 ± 1.7	0.15 ± 0.01	0.08 ± 0.01	64.5 ± 3.7	7 ± 2
3tC-200 nm/s	4.5 ± 0.5	3.2 ± 0.5	7.7 ± 0.7	33.6 ± 2.9	0.13 ± 0.03	0.10 ± 0.03	58.3 ± 7.8	17 ± 3
Unmodified-150 mM Na^+^	6.6 ± 0.4	3.4 ± 0.4	9.9 ± 0.6	43.2 ± 2.6	0.15 ± 0.04	0.08 ± 0.02	66.2 ± 6.1	36 ± 5

^a^ ${{X}_{B \to TS}}$ , ${{X}_{S \to TS}}$, ${{X}_{BS}} = {{X}_{B \to TS}} + {{X}_{S \to TS}}$ are the elongations during the transition from the B- to the transition state (TS), from TS to the S-state, and the total elongation from B- to S-state using the Bell-Evans model.

^b^Number of bps estimated using $n = X/{{x}_1}$ and dividing these extensions by the number of bps (Equation S13) in the transition yields the extension per bp that is constant within our error of measurement for all studied duplexes.

^c^The transition state lies at ∼60% extension from B- to S-states.

^d^N - total number of transitions per individual stretch/release cycle averaged for a given dsDNA molecule. The reported values are the mean ± SEM. Data were measured at 1 M NaCl and 50 nm/s unless indicated. Unmodified and 3tC duplexes were also measured at 1 M and 200 nm/s pulling rate and 150 mM NaCl and 50 nm/s pulling rate. Note: The upper-case values, including length and free energies, are for the n-bp DNA duplex while all lower-case values are per bp. Also, G with subscripts denotes a free energy, while the use of an arrow in the subscripts refers to a transition state free energy.

As observed for the total duplex extension during the B-to-S transition, ${{X}_{BS}},$ the extensions to $({{X}_{B \to TS}})$ and from $({{X}_{S \to TS}})$ its transition state appear to be proportional to the number of bps undergoing the transition, as follows from the constant value of these extensions per bp for all studied DNA duplexes: ${{x}_{B \to TS}} = 0.14 \pm 0.01\ nm/bp$ and ${{x}_{S \to TS}} = 0.09 \pm 0.01\ nm/bp$ (see Table 2). The transition state is located ∼60% from the B-DNA and 40% from S-DNA extension. All bps in the B-DNA duplex deform similarly under the action of axial tension and the duplex needs to be stretched 60% towards the S-DNA state prior to spontaneous further elongation into the S-state. The measured transition rates at ${{F}_{tr}}$, ${{k}_{B \to S}}( {{{F}_{tr}}} ) = {{k}_{S \to B}}( {{{F}_{tr}}} )$ appear almost the same for all studied dsDNA oligonucleotides (Figure 3C, Supplementary Figure S1M-S1R and Supplementary Table S4). The Bell–Evans fitting allowed us to estimate the zero-force transition rates, $k_{B \to S}^0$, $k_{S \to B}^0$ (Equation S11, which is the combination of the attempt rate and probability of overcoming the transition barrier) reported in Supplementary Table S5. However, the Bell-Evans type analysis does not allow us to estimate the height of the transition barriers between the B- and S-states at zero force for the B-to-S or S-to-B processes, ${{G}_{B \to TS}}$ and ${{G}_{S \to TS}}$, and the universal attempt rate $k_{BS}^0$ (as introduced in Equation S11).

A major goal of the kinetic analysis of any two-state transition is to reconstruct the free energy profile in the absence of force (54). This is useful for understanding if the transition can occur without force, how long it may take, for how long the higher free energy state can last, and the force required for it to occur on a particular time scale. Our FDCs contain additional kinetic information that can potentially be used to obtain the transition attempt rate, $k_{BS}^0$, and transition free energy barrier heights, ${{G}_{B \to TS}}$ and ${{G}_{S \to TS}}$, individually. Thus, we attempted to use the approach proposed by Dudko *et al.* (40,52,58–60) fitting the shape of the transition force probability distributions for the forward and reverse transitions versus force, $\rho ( {B,\ F} )$ and $\rho ( {S,F} )$, shown in Figure 3A. However, this approach did not work, as in our case the $\rho ( {B,F} )$ and $\rho ( {S,F} )$ almost overlap and do not contain enough information on the transition barriers, as the transition is fast and occurs almost in equilibrium on the time scale of our stretch-and-release cycles (∼4 s).

### dsDNA stretching at higher pulling rate reports on the same B-to-S transition thermodynamics and kinetics

To investigate the B-to-S transition further away from equilibrium, we pulled our DNA oligonucleotides faster (at 200 nm/s instead of 50 nm/s, see Supplementary Figure S4A, B for FDCs) in an attempt to obtain more information on the transition kinetics. At such a high pulling rate only very few transitions occurred over ∼1s duration of the stretch/release cycle (see last column of Table 2), leading to poorer statistics of the transitions. However, performing enough experiments allowed us to conclude that the 4-fold increase of the pulling rate had practically no effect on the apparent transition thermodynamics or kinetics. The reason that faster pulling did not lead to any measurable changes in the probability distribution and rates of the B-to-S and S-to-B transitions (compare Supplementary Figure S1G, M with Supplementary Figure S4E, I for Unmodified and Supplementary Figure S1J, P with Supplementary Figure S4F, J for 3tC) is that the duplex length changes associated with each process ${{X}_{B \to TS}}$ and ${{X}_{S \to TS}}$ are large, ∼4–6 nm (see Table 2 and Supplementary Figure S3B). As the transition rates ${{k}_{B \to S}}( F )$ and ${{k}_{S \to B}}( F )$ depend exponentially on the product $F \cdot {{X}_{B \to TS}}$ or $F \cdot {{X}_{S \to TS}}$ (see Equation S9), large changes of the transition rates matching our increased pulling rate lead to unmeasurably small changes in the transition force. In other words, the B-to-S transition data obtained at 4-fold faster DNA pulling reports on the same equilibrium and kinetic properties of the transition. Specifically, we obtain the same equilibrium transition force and free energy, and the same scaling of these parameters with the number of bps going through the transition. We also confirm the transition barrier positioned at 60% extension beyond B-DNA toward S-DNA, and the scaling of these extensions and transition barriers with *n* (see data in Table 2, Supplementary Figure S4I, J and Supplementary Table S4, Supplementary Table S5). The data at higher pulling rate reports on the same ∼5–6 k_B_T stabilization of the central part of the dsDNA oligonucleotide by three tC modifications (see Table 1). The only parameter that changed significantly between the 50 and 200 nm/s pulling rate was the average total number of B-to-S transitions over each stretch/release cycle, *N* (see Table 2), which decreased proportionally to the reciprocal rate of stretching, i.e. proportionally to the net time of the stretch/release cycle. As is shown in Table 2, the total average number of the transitions *N* for the unmodified duplex decreased from 25 ± 3 to 7 ± 2 as the pulling rate increased 4-fold. Similarly, for the 3tC duplex *N* decreased from 65 ± 6 to 17 ± 3 with the same 4-fold rate increase, strongly suggesting that the same transition kinetics was observed at both rates, and the number of transitions is reflective of the average transition rate at the transition force. Below we will use sets of data obtained at both pulling rates to reconstruct the free energy profile of the B-to-S transition.

### Effect of salt on the B-to-S transition

All data discussed so far was obtained in 1 M NaCl to promote S-DNA formation over DNA melting. To learn more about the B-to-S transition under conditions closer to physiological salt we repeated our dsDNA stretching experiments in 150 mM NaCl solution. At this lower salt, it was difficult to record FDCs, since in many FDCs the dsDNA oligonucleotides melted instead of undergoing a B-to-S transition, and the tether was lost. The B-to-S transition was still observed in most FDC of the Unmodified DNA (Supplementary Figure S4C,G), but for the 3tC DNA a very limited number of stretch/release cycles (Supplementary Figure S4D,H) with multiple B-to-S transitions were observed and the results of the analysis of all collected data is reported in Table 1, Table 2, Supplementary Figure S3B, Supplementary Figure S4, and Supplementary Table S6, respectively. Overall, the results on the B-to-S transition at 150 mM NaCl can be summarized as follows: lower salt leads to more dsDNA oligonucleotide end fraying and fewer bps undergoing the B-to-S transition, leading to a smaller transition extension $\Delta X$ (by ∼0.6 nm = 3 bps for Unmodified and by ∼1.2 nm = 5 bps for 3tC) and a smaller transition free energy ${{G}_{BS}}$ (by ∼13 k_B_T for Unmodified and by ∼19 k_B_T for 3tC) (see Table 1 and Supplementary Table S6). Further analysis suggests that the transition free energy still scales with the number of bps undergoing the B-to-S transition. While the dsDNA extension $\Delta X$ and the transition barrier distance from the B- $({{X}_{B \to TS}}$) and S-state (${{X}_{S \to TS}}$) decrease, they still scale with the number of bps undergoing the B-to-S transition. The Bell-Evans analysis shows that the transition state is located at the same position at ∼60% extension from the B- towards the S-state as in high salt (Table 2, Supplementary Figure S4K, L and Supplementary Table S4, Supplementary Table S5). The measured per bp B-to-S transition free energy in 150 mM NaCl, $g = 3.50 \pm 0.01$ (for Unmodified dsDNA) is just slightly lower than the same quantity $g = 3.56 \pm 0.01$ in 1 M NaCl (Table 1). This decrease in the transition free energy is somewhat smaller than might be expected based on polyelectrolyte theory, as discussed in Supplementary Section 9 (Equations S21–22). We also observe an increase in the total average number of the B-to-S transitions over the stretch/release cycle for the Unmodified duplex from 25 ± 3 in 1 M NaCl to 36 ± 5 in 150 mM NaCl (Table 2) and 3tC from 65 ± 6 in 1 M NaCl to 74 ± 7 in 150 mM NaCl due to the slight decrease in the transition free energy barrier per bp ${{g}_{TS}}( {150\ mM\ NaCl} ) = 0.07$ compared to ${{g}_{TS}}( {1\ M\ NaCl} ) = 0.11$ as discussed in Supplementary Section 9 (Equation S23, and Supplementary Table S6). We conclude that both the thermodynamics and kinetics of the B-to-S transition are only slightly affected by lowering the salt from 1 M to 150 mM NaCl. A more detailed data analysis and discussion of the salt effect on the B-to-S transition is presented in Supplementary Section 9.

### The number of bps undergoing the B-to-S transition depends on the transition force

**Figure 4.**
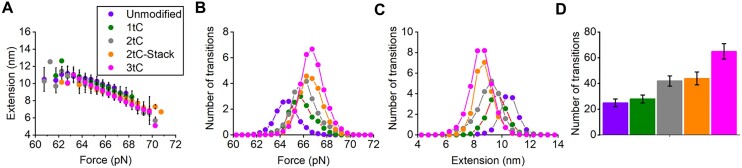
The free ends of the stretched dsDNA oligonucleotides fray progressively at higher forces prior to the cooperative B-to-S transition in the rest of the molecule. (**A**) dsDNA length changes during the transition as a function of transition force. (**B**) Average number of transitions during stretch-release cycle as a function of transition force. (**C**) Number of transitions as a function of extension. (**D**) Average number of transitions per stretch/release cycle for different oligonucleotides. Unmodified (violet), 1tC (green), 2tC (grey), 2tC-Stack (orange) and 3tC (magenta). Data obtained at 50 nm/s pulling rate at 1 M NaCl.

So far, we have analyzed the B-to-S DNA transition thermodynamics and kinetics using the average characteristics of the transition. However, the individual B-to-S transitions during each stretch/release cycle do not in general occur at the average ${{F}_{tr}},$ but are distributed within ± 4 pN of the probability distribution centered at ${{F}_{tr}}$, as presented in Figure 4B. Similarly, the dsDNA extensions are not all equal to the average ${{X}_{BS}}$, but are rather distributed around this value ± 2 nm (see Figure 4C). Moreover, the instantaneous transition force and extension are not random, but are strongly correlated with each other, with higher transition forces leading to smaller duplex extensions for Unmodified and tC modified duplexes, as shown in Figure 4A. An increase in the transition force by 1 pN leads to a shortening of the duplex extension during the transition by 1 nm, equivalent to ∼4 fewer bps undergoing the B-to-S transition. This result is consistent with higher transition forces leading to more bps fraying, and fewer bps undergoing the B-to-S transition, leading to a smaller transition extension. The universal nature of this dependence is confirmed by the fact that the slope of the ${\mathrm{\Delta }}X( F )$ dependence for the individual dsDNA oligomer (see Figure 4A), $\frac{{d\Delta X}}{{dF}} \approx 1\frac{{\ nm}}{{pN}}$, appears to be the same as the shift in the average ${\mathrm{\Delta }}X$ with average ${{F}_{tr}}$ for the different dsDNA constructs. For example, as presented in Table 1 and in Figure 4A, the extension during the B-to-S transition in the tC-modified oligomers has an average ${{F}_{tr\ }}$ that is 2.5 pN higher than the Unmodified DNA duplex, and an average elongation that is 2 nm smaller, showing the same $\frac{{d\Delta X}}{{dF}}$ slope as for individual DNA constructs. The ${\mathrm{\Delta }}X( F )$ curves for individual dsDNA duplexes are parallel to each other and shifted to higher forces, proportionally to the shift of the average ${{F}_{tr}}$.

In addition to the transition elongation, the B-to-S transition frequency also depends on force. Specifically, the number of transitions (proportional to the transition rate) versus force for all duplexes has a maximum at ∼${{F}_{tr}}$ (see Figure 4B). This maximum, as well as the total transition number, *N*, during each stretch-and-release cycle (integral of the number of transitions versus force in Figure 4B presented in Figure 4D and in Table 2), clearly increases with the number of tC modifications. Likewise, the extension at which the maximum number of transitions occurs decreases with the number of tC incorporations (Figure 4C). Similar results were observed for the base rearranged duplexes (Supplementary Figure S5). As the duration of each stretch-and-release cycle for all dsDNA duplexes stretched at the same pulling rate was the same, *N* is proportional to the true average B-to-S transition rate in the vicinity of ${{F}_{tr}}$. Similar results from corresponding experiments at higher pulling rate of 200 nm/s and in lower salt (150 mM NaCl), are reported in Supplementary Figure S6. The duplex extension between the B- and S-states decreases with increasing force and the extension is smaller at higher pulling rate and lower salt for both Unmodified (Supplementary Figure S6E, I) and 3tC (Supplementary Figure S6M, Q). The transition distributions versus force and extension after normalizing to the total number of transitions are very similar between the two pulling rates (Supplementary Figure S6F, G, J, K) and the two salt concentrations (Supplementary Figure S6N, O, R, S).

### Reconstructing the B-to-S free energy profile

**Figure 5.**
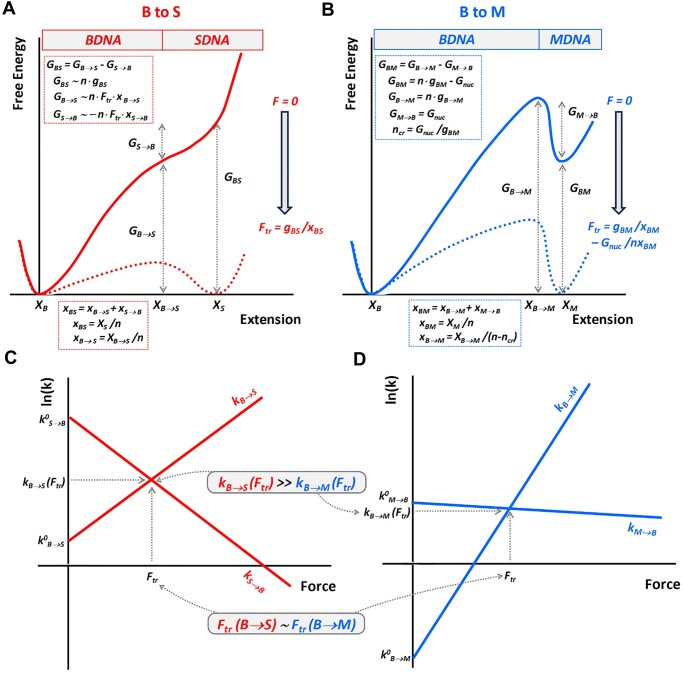
Free energy profiles versus extension of the *n* bp dsDNA oligonucleotide transitioning from B-DNA to either S- (panel **A**), or M- (melted, panel **B**) state. Continuous lines correspond to the zero-force free energy profile, dashed lines to the free energy profile at the transition force ${{F}_{tr}}$. Two bottom panels **C** and **D** present the force dependence of the forward and reverse rates for the B-to-S (**C**) and B-to-M (**D**) transitions consistent with the corresponding free energy profiles in panels **A** and **B**.

Our data also contain information on the average B-to-S transition rates and their dependence on the number of bps undergoing the transition. The total number of forward and reverse B-to-S transitions during each stretch/release cycle (*N*) is different for the seven dsDNA oligonucleotides, and depends systematically on the average number of bps, *n*, undergoing the transition (see last column in Table 2 and Figure 4D). Assuming that the transition barrier free energy scales with *n*, as do all other characteristics of this transition, allows us to obtain the per bp transition free energy barrier, ${{g}_{TS}}( {{{F}_{tr}}} ) \approx 0.11,$ at the transition force ${{F}_{tr}}$ (described in Supplementary Section 7 and Equation S16). This is a very low transition barrier with a free energy much smaller than the equilibrium free energy of the transition, $g,$$\frac{{{{g}_{TS}}( {{{F}_{tr}}} )}}{g} = 0.03 \ll 1.$ The free energy profile of the B-to-S transition for the n-bp duplex is presented schematically in Figure 5A. The dotted red line shows the B-to-S transition free energy profile at ${{F}_{tr}}$ with two shallow minima at the B- and S-extensions separated by a low barrier ${{g}_{TS}}( {{{F}_{tr}}} )$ located at ∼60% extension from B- toward S-DNA, and a steep free energy increase near the B-DNA and beyond the S-DNA extensions.

Indeed, B-DNA is known to have significant stiffness beyond its contour length until ∼ 50 pN, i.e., until its 5% elongation due to the known high elastic modulus ∼1000 pN (4,34). The 1.7-fold longer S-DNA is even more stiff beyond its contour length and stretches with an elastic modulus of ∼3000 pN (26). Furthermore, knowing the transition free energy profile at the transition force allows to estimate the transition barrier per bp in the absence of duplex stretching force, ${{{\boldsymbol{g}}}_{{\boldsymbol{TS}}}}( {{\boldsymbol{F}} = 0} ),$ by simply adding the term ${{{\boldsymbol{F}}}_{{\boldsymbol{tr}}}} \cdot {\boldsymbol{x}}$, i.e. ${\boldsymbol{g}}( {{\boldsymbol{F}} = 0} ) = {\boldsymbol{g}}( {{{{\boldsymbol{F}}}_{{\boldsymbol{tr}}}}} ) + {{{\boldsymbol{F}}}_{{\boldsymbol{tr}}}} \cdot {\boldsymbol{x\ }}$to the free energy profile at ${{{\boldsymbol{F}}}_{{\boldsymbol{tr}}}}$ (see a more detailed discussion in Supplementary Section 8 and Equation S17). The resulting free energy profiles of the B-to-S transition for the n-bp long duplex at ${\boldsymbol{F}} = 0,$${\boldsymbol{g}}( {{\boldsymbol{F}} = 0} ){\boldsymbol{\ }}$is presented in Figure 5A as a solid red line. The zero force B-to-S transition barrier (Supplementary Table S7) was estimated as described in Supplementary Section 8. We conclude that S-DNA does not have even a metastable free energy minimum in the absence of force, i.e. it is an entirely force-induced state. This is very different from the free energy profile of the B-DNA melting transition (i.e. B-to-M transition) presented for comparison in Figure 5B and discussed below.

### The attempt rate for the B-to-S transition is slow compared to the attempt rate for DNA melting

Finally, the estimate of the B-to-S transition barrier free energy allows us to also estimate its attempt rate, $k_{BS}^0$, which is a universal rate constant for the B-to-S transition that is independent of the applied force, duplex length, or stability. Due to the approximate nature of these results $k_{BS}^0$ can only be determined up to an order of magnitude, which is a significant result, considering that it is the first experimental determination of this parameter. To estimate $k_{BS}^0$ we can use our experimental result that at ${{F}_{tr}}$ the B-to-S transition rate is ${{k}_{B \to S,n}}( {{{F}_{tr}}} ) = k_{BS}^0 \cdot {{e}^{ - n \cdot {{g}_{TS}}}}\sim 1 - 10{{s}^{ - 1}}$. Therefore, $k_{BS}^0\sim{{k}_{B \to S,n}}( {{{F}_{tr}}} ) \cdot {{e}^{n \cdot {{g}_{TS}}}}\sim( {1 - 10} ){{s}^{ - 1}} \cdot {{e}^{4.7}}\sim{{10}^2} - {{10}^3}\ {{s}^{ - 1}}$. Here, we used $n = 47$, and ${{g}_{TS}}\sim0.1$ for the Unmodified dsDNA. We conclude that the universal B-to-S transition attempt rate independent of the duplex stability and length is:


\begin{equation*}k_{BS}^0 \sim {{10}^2} - {{10}^3}{\mathrm{\ }}{{s}^{ - 1}}\end{equation*}

This B-to-S transition attempt rate $k_{BS}^0\sim{{10}^2} - {{10}^3}\ {{s}^{ - 1}}$ is slow compared to the known melting transition attempt rate $k_{BM}^0\sim{{10}^5} - {{10}^6}\ {{s}^{ - 1\ }}$ (61). However, the B-to-S transition at the transition force is typically much faster than B-DNA melting (as discussed below) due to the extremely low B-to-S transition barrier.

## Discussion

### Equilibrium B-to-S and B-to-M transition free energies are similar

Previous experiments on long polymeric dsDNA (17–19) have demonstrated that the overstretching of dsDNA can proceed either via B-to-S or B-to-M transitions or a combination of the two, depending on solution conditions and DNA composition (26,29–32). Co-existence of these two types of overstretched DNA, and the similarity of their stabilities and extensions, remains intriguing. The biological role of dsDNA melting, or strand separation, is well appreciated in many cellular processes, such as transcription bubble formation, single stranded DNA-protein binding, DNA supercoiling–induced promoter opening, and CRISPR-Cas9 DNA editing. In contrast, the biological role of S-DNA remains largely unknown. In the present work, we were able to quantify the equilibrium free energy, 3.6 k_B_T/bp, of the pure B-to-S transition, which is surprisingly similar to the average per bp free energy of melting of a B-DNA oligonucleotide with ∼70% GC composition under identical salt and temperature conditions (1 M NaCl, 25°C) (17,18,20,22,62). Our estimate of the B-to-S per bp transition free energy is ∼1 k_B_T higher than previously estimated, assuming that all bps in a 60-mer oligonucleotide would undergo the B-to-S transition (63).

### tC modifications stabilize B-DNA relative to S-DNA

The increase in stability of the central part of the dsDNA duplex by introducing one, two or three tC modifications leads to an increase in the B-to-S transition force, causing more bps to fray prior to the B-to-S transition and fewer bps to go through the transition. The relatively minor stabilization (∼5.4 k_B_T) of the central part of the dsDNA oligonucleotide upon introduction of 3 tC modifications leads to a ∼2.5 pN increase in the transition force, resulting in 9 fewer bps undergoing the B-to-S transition and a reduction of the equilibrium transition free energy by ∼25 k_B_T. Comparing B-DNA stabilization by one, two or three tC modifications (Figure 2D) we conclude that each tC increases the B-to-S transition free energy by ∼2 k_B_T (except for the first tC modification that stabilizes B-to-S by ∼3k_B_T), and that placing two tC modifications adjacent to each other in the same strand has the same stabilizing effect on B-DNA as two independently and distantly positioned tC modifications. This result is similar to the observed stabilization of B-DNA by tC with respect to DNA unzipping (i.e. melting) of ∼1.3 k_B_T (50). It is also in agreement with the dsDNA *T_m_* shift induced by these tC modifications (49,50). We conclude that the tC modifications have a similar stabilizing effect on the B-to-S and B-to-M transitions.

We further explored the effect of reducing the strength of stacking of subsequent purine bases on the B-to-S transition, hypothesizing that it will lead to lower B-to-S transition free energy. Indeed, we observed slight duplex destabilization with respect to the B-to-S transition in the original duplex by ∼4 k_B_T. Yet this is a minor effect, considering that 13 pairs of subsequent purine bases were replaced in the dsDNA oligonucleotide (Figure 2H and Table 1). This contrasts with the free energy of thermal melting calculated using DINAMelt that showed duplex stabilization by 6.9 k_B_T for Unmodified-Low compared to Unmodified (Supplementary Table S3). Hence, the same dsDNA sequence modification that stabilizes B-DNA with respect to melting also destabilizes it with respect to the B-to-S transition.

### Kinetic properties of the B-to-S transition

The B-to-S transitions occurred ∼10–100 times within a narrow force range of only ∼3 pN around the equilibrium transition force, showing that the two states are almost in equilibrium. However, we observe that the B-to-S transition occurs ∼2.5-fold faster for 38 bp dsDNA compared to 47 bp dsDNA. This allows us to estimate the per bp transition barrier free energy at ${{F}_{tr,}}$ which is very low, ∼0.1 k_B_T/bp, and is positioned at a dsDNA extension approximately 60% along the path between the B- and S-DNA forms. We conclude that the B-to-S transition is reasonably fast, with a rate between 1 and 10 s^−1^, and only weakly dependent on dsDNA length. This conclusion is in remarkably good agreement with the results of the only other lab that studied kinetics of the B-to-S transition in polymeric dsDNA, observing ∼25 bp-long cooperative dsDNA fragments undergoing a B-to-S transition at a rate of ∼3 s^−1^ at the overstretching force, with the transition barrier positioned approximately midway between the B- and S-states (34,35). Despite the fact that the cooperative duplex length in polymeric DNA of ∼25 bps is substantially shorter than the range of the duplex length (38–47 bps) studied here, and the solution conditions (25°C, 150 mM NaCl, 10 mM Tris, 1 mM EDTA, pH 8.0) in these λ-DNA experiments (34,35) were slightly different from ours (23°C, 1 M NaCl and 150 mM, 10 mM Tris, 1 mM EDTA, pH 7.4), the B-to-S transition rates for the duplexes studied here and polymeric λ-DNA are similar. This result is consistent with only a weak dependence of the B-to-S transition rate on the DNA duplex length and stability, due to the extremely low per bp transition barrier at the transition force ${{F}_{tr}}$ (${{g}_{TS}}( {{{F}_{tr}}} )\sim0.1\ {{k}_B}T$). Such a low barrier at ${{F}_{tr}}$ implies that the B-to-S transition free energy profile $g( {F = 0,x} ){\mathrm{\ }}$ in the absence of force (Figure 5A) is an increasing function of extension without a metastable free energy minimum at the S-DNA extension. Thus, S-DNA is an entirely force-induced state. Our analysis of the B-to-S kinetics versus dsDNA oligonucleotide bp number *n* in this study suggests that the DNA duplex is quite elastic at all extensions beyond a few percent of the B-DNA contour length and all the way to the S-DNA contour length. At a few percent beyond the B-DNA contour length and up until ∼ 60% on the path towards S-state, the dsDNA stretches easily at ∼${{F}_{tr}}$ with an elastic modulus of just a few pN without transitioning to the S-state, and ‘snaps’ into the force-stabilized S-DNA beyond this transition state extension. These conclusions are qualitatively summarized in the sketch of the B-to-S free energy profile at zero force and at the transition force in Figure 5A. Our measurements of the B-to-S rate $( {{{k}_{B \to S,n}}( {{{F}_{tr}}} )\sim1 - 10\ {{s}^{ - 1}}} )$ and the per bp transition barrier height (∼0.1 k_B_T) allow us to estimate the universal attempt rate for overcoming the B-to-S transition barrier (introduced in Equation S11) to be $k_{BS}^0\sim{{10}^2} - {{10}^3}\ {{s}^{ - 1}}$.

### B-to-S transition and force-induced melting have very different kinetics

As the B-to-S transition kinetics are similar for dsDNA oligonucleotides and cooperative segments of polymeric dsDNA (34), the faster B-to-S kinetics can be a deciding factor in selecting the B-to-S pathway over the slower B-to-M overstretching pathway for polymeric dsDNA subject to fast stretching by physiological forces in the cell (64). It is, therefore, important to compare and contrast the B-to-S transition kinetics characterized in this work with the B-to-M kinetics in dsDNA oligonucleotides characterized previously in thermal melting studies (28,65–67), as well as using force spectroscopy (31,32,68,69). From dsDNA oligonucleotide thermal melting studies, we know that the entire duplex, except for its last ∼2–3 bps, must melt prior to complete strand separation. Also, the reverse process of DNA oligonucleotide strand annealing requires duplex nucleation via formation of the same few bp, followed by a spontaneous ‘zipping’ of the rest of the duplex (27,70,71). This nucleation event is associated with only a minor DNA length change, and has an unfavorable nucleation free energy ${{G}_{nuc}}$ of the two DNA strands losing their entropy upon their initial attachment, as well as overcoming the electrostatic repulsion between the two negatively charged strands (27,72). ${{G}_{nuc}}$ also grows with temperature, as more of the less stable base pairs are needed to nucleate annealing of the two strands at higher temperature. Thus, ${{G}_{nuc}}$ grows with decreasing concentration of complementary DNA strands, as well as in lower salt and at higher temperatures, but is practically independent of force and DNA duplex length. ${{G}_{nuc}}$, in turn, determines the DNA strand annealing rate: ${{k}_{BM}}( {{{F}_{tr}}} )\sim k_{BM}^0 \cdot {{e}^{ - {{G}_{nuc}}}}$, where $k_{BM}^0\sim{{10}^5} - {{10}^6}\ {{s}^{ - 1}}$ is the melting attempt rate (61,73,74). At high salt and high strand concentration conditions, the duplex nucleation barrier is low, and DNA strand annealing can be as fast as, or even faster, than the B-to-S transition. In the opposite case of low local dsDNA strand concentration, lower salt, or higher temperature, the duplex nucleation free energy becomes high, making the strand annealing rate very slow (32) and reducing the force does not make it faster. This is in contrast to the dsDNA oligonucleotide strand separation rate, which can be strongly facilitated even by a very small increase in force, such that even at very high pulling rates, the apparent melting force appears to be very close to its equilibrium value for a sufficiently long dsDNA oligonucleotide (32,68,69). This is because dsDNA oligonucleotide melting requires almost all bps to melt at once, leading to the large DNA elongation associated with the strand separation rate. This very strongly force-dependent melting rate ${{k}_{B \to M}}$ is presented in Figure 5D. At the transition force the free energies of the B- and M-states are equal, as are the transition barriers between both states. Therefore, the nucleation transition free energy barrier (${{G}_{M \to B}} = {{G}_{nuc}})$ at ${{F}_{tr\ }}$ is practically unaffected by force and remains like the transition barrier without force. This barrier is determined by the nucleation free energy and is positioned very close to the M-DNA state, as sketched in Figure 5B. Moreover, since the nucleation free energy is not affected either by the force or by the dsDNA length, the height of the transition barrier as well as the transition rate $({{k}_{M \to B}})$ are also force- and dsDNA length-independent (see Figure 5D). The melting rate at the transition force ${{k}_{B \to M}}( {{{F}_{tr}}} ) = {{k}_{M \to B}}( {{{F}_{tr}}} )$ is determined by the force-independent annealing rate ${{k}_{M \to B}}$, which becomes exponentially slower with increasing nucleation barrier ${{G}_{nuc}}.$ This was, indeed, observed in the experiments by Bosaeus *et al.*, (32), when an AT-rich dsDNA oligonucleotide with its two strands crosslinked either at one, or the other, or both ends was force-melted in a solution of 1 M or 5 mM NaCl salt. While the doubly crosslinked dsDNA in 1 M salt melted and re-annealed in a fast and reversible manner with a rate of ∼1000 s^−1^, the same dsDNA with its strands crosslinked only at one end, or stretched in low salt, melted at a rate <1 s^−1^ and annealed yet slower, also showing profound stretch-release hysteresis (32). These results highlight the fundamental difference between the kinetics of the force-induced dsDNA melting and the B-to-S transition. According to this study, the B-to-S transition in dsDNA duplexes of variable length can be made arbitrarily fast by minor variations of the applied force from its equilibrium transition value, such that the B-to-S transition appears to be fast and almost in equilibrium in a broad range of pulling rates. In contrast, re-annealing of the DNA strands cannot be made faster than its rate typical for the specific solution conditions by any force reduction. Comparison of the free energy profiles, as well as of the rates versus force dependencies for B-to-S and B-to-M transitions, are presented in Figure 5A–D. We conclude that the B-to-S transition differs from the B-to-M transition primarily kinetically.

### Modified bases can be used to probe local nucleic acid structure

It is important to stress the use of modified bases to slightly vary the local structure and properties of nucleic acids. Since the optical tweezers instrument used here allows studies of short oligonucleotides, the modified bases can be positioned anywhere in the structure with base-pair resolution. They can be used to modify nucleic acids in a large variety of ways. We have recently demonstrated how this can locally modify the stability and structure of DNA and RNA hairpins (50,75). In the current study, we demonstrate how tC slightly increases the stability of B-DNA compared to S-DNA and how this in turns allows us to shift the overstretching transition slightly and, in that way, massively increase the information we obtain from the system without varying the DNA sequence.

## Conclusion

Based on the findings of this work, we expect that in most situations, including physiological conditions, the B-to-S transition will occur faster than melting for dsDNA oligonucleotides as well as for segments of polymeric dsDNA upon fast stretching. Because of this difference in rates, it is possible that the same long dsDNA molecule may overstretch via the internal melting pathway at lower pulling rates and via the B-to-S transition pathway at higher pulling rates. Therefore, S-DNA may provide a faster-forming intermediate state between B-DNA and locally M-DNA. Alternatively, it is possible that S-DNA can be captured and stabilized by specific enzymes able to recognize this overstretched DNA state when it encounters short force pulses during biological processes.

## Supplementary Material

gkae1183_Supplemental_File

## Data Availability

Data are available upon reasonable request to the corresponding authors.
