# Supplementary material for: Force-induced melting and S-DNA pathways for DNA overstretching exhibit distinct kinetics
Source: Nucleic Acids Res. 2024 Dec 9;53(1):gkae1183. doi: 10.1093/nar/gkae1183 (PMC11724298; doi:10.1093/nar/gkae1183)
Supplement: gkae1183_Supplemental_File [file gkae1183_supplemental_file.pdf]

Supplementary Data for:

Force-induced melting and S-DNA pathways for DNA overstretching exhibit distinct kinetics

Vinoth Sundar Rajan[1,2], Sune Levin[1], Micah J. McCauley[3], Mark C. Williams[3], Ioulia Rouzina[4], L. Marcus Wilhelmsson[2*], Fredrik Westerlund[1*]

[1]Department of Life Sciences, Chalmers University of Technology, Gothenburg, Sweden.
[2]Department of Chemistry and Chemical Engineering, Chalmers University of Technology, Gothenburg, Sweden.
[3]Department of Physics, Northeastern University, Boston, MA, USA.
[4]Department of Chemistry and Biochemistry, The Ohio State University, Columbus, OH, USA.

*Email: marcus.wilhelmsson@chalmers.se, fredrikw@chalmers.se

TABLE OF CONTENTS

Supplementary Materials and Methods

1. Beads preparation
2. Optical tweezers setup
3. Calculation of the equilibrium transition force and extension
4. Free-Energy Calculation
5. Estimating the effects of modifications on duplex stabilization
6. B-to-S transition kinetic analysis
7. Reconstruction of the B-to-S transition free energy profile at the transition force, F_{tr}.
8. Reconstruction of the B-to-S transition free energy profile in the absence of DNA stretching force, *F=0*.
9. Effect of salt on the B-to-S transition

Supplementary Figures

Supplementary Tables

Supplementary References

Supplementary Materials and Methods:

1. **Beads preparation**

Polystyrene beads coated with streptavidin were purchased from Kisker Biotech (Ref. #: PC-S-2.0; 2.0 to 2.9 mm diameter). Anti-digoxigenin beads were prepared in-house from Protein G-coated polystyrene beads (Kisker Biotech, Ref. #: PC-PG-3.0; 3.0 to 3.4 mm diameter). 1.0 mL of Protein G-coated beads was washed twice with linking buffer (100 mM Na_2HPO_4, 100 mM NaCl, pH 8.5). Resuspended beads were incubated with 120 µl of anti-digoxigenin polyclonal antibodies (Roche, Ref. #: 11333089001) and 60 µL of cross-linker dimethyl pimelimidate (Thermo Fischer, Ref. #: 21666). After incubation, the anti-digoxigenin coated beads were washed twice with PBS buffer (140 mM NaCl, 2.7 mM KCl, 61 mM K_2HPO_4, 39 mM KH_2PO_4, 0.02% NaN_3, pH 7.0) and resuspended in 1 ml of PBS buffer. The dsDNA was incubated with digoxigenin coated beads for 30 min at room temperature and diluted in 1.0 mL of experimental buffer. The streptavidin-coated beads were diluted in experimental buffer before experiments.

2. **Optical tweezers setup**

The experiments were performed using an in-house built optical tweezers instrument described in detail in Bosaeus et al., (1). Two counter-propagating 150 mW, 845 nm diode lasers were used to form a single optical trap within the microfluidic chamber mounted on a motorized stage. The fluidic chamber is divided into three channels; two of them are used to dispense the two different types of coated beads and the third channel contains the trap and the micropipette. The optical trap is used to capture one of the polystyrene coated beads and the other coated bead is immobilized by suction at the tip of the micropipette. Optical fibers are used to guide the laser beams, and the position of the trap is measured by redirecting 5% of the light intensity onto a position sensitive detector (PSD). The remaining light is focused through a water-immersion objective lens (60X, NA 1.20) to form the trap. The light exiting the trap is collected by an identical objective lens and redirected to a PSD and photodiode to measure the forces acting on the trapped bead in all three dimensions. A quarter-wave plate and a polarizing beam splitter are used to redirect the light exiting the condensing objective lens to the force detector by turning the polarization of light 90^0 relative to the light entering

the objective lens The forces are measured based on the conservation of light momentum (2,3).

3. Calculation of the equilibrium transition force and extension

The force-distance curves obtained from the measurements are analyzed using custom-made MATLAB programs. The bistability of the transitions suggests a two-state process and the transition force was calculated based on this assumption (1). The data points are assigned to either the B- or S-state depending on the proximity to straight lines fitted to the forces below the transition range (B-form fit) and above the transition range (S-form fit) (Figure 1C, zoom). The probability of finding the molecule in any of the two states is assumed to follow a Boltzmann distribution function and can be obtained by counting the data points for the S- (N_S) and B-state (N_B), respectively, for a bin size of 0.5 pN. The overstretched probability P_S was calculated and fitted to a two-state model,

$$P_S(F) = \frac{N_S}{N_S+N_B} = \frac{1}{(1+e^{b-Fa})} \quad \text{(S1)}$$

The equilibrium transition force F_{tr} can be found as the force at which the probability for being in both states is the same: $P_S = \frac{1}{2}$, and is defined by the fitted parameters a and b, as $F_{tr} = \frac{b}{a}$. The extension (length change between the B- and S- states) of the dsDNA at F_{tr} was measured as the change in force divided by the slope of the B-form fit (K_{eff}). We estimate that the extension (ΔX) calculated represents only the length change between the B- and S -states and that the contributions from the extension of the handles and the bead position relative to the trap are negligible.

4. Free-Energy Calculation

The equilibrium transition free energy, G_{BS}, was determined in several complimentary ways. The simplest way is:

$$G_{BS} = F_{tr}\Delta X \quad \text{(S2)}$$

This expression is the non-equilibrium work done by the instrument on the dsDNA oligonucleotides. The reason that the calculated work provides a good estimate of the equilibrium transition free energy is that the B-to-S transition in this set of experiments is, in

fact, occuring very close to equilibrium. Indeed, there are 30-100 transitions that typically occur over each of the stretch-release cycles, and the probability distributions of the forces of the forward and reverse transitions almost overlap (see Figure 3A and Figure S1 G-L). The G_{BS} values estimated according to Equation S2 for all dsDNA duplexes studied are shown in Table 1. Also, several methods were used to extract the free energy between the B- and S-states from work distributions such as Crook's fluctuation theorem, Jarzynski's equality and Bennett's acceptance ratio (4-6). The acceptance ratio method give more accurate results by averaging over all the measured work when the distributions have little or no overlap. The work is weighted by a special function (f_x) and then averaged,

$$Z_{S\to B}(x) - Z_{B\to S}(x) = \frac{G_{BS}}{k_B T}$$

$$Z_{B\to S}(x) = ln < f_x(\mathrm{W}) \exp\left(\frac{-W}{k_B T}\right) >_{B\to S}, \qquad Z_{S\to B}(x) = ln < f_x(\mathrm{W}) >_{S\to B}$$

$$f_x(W) = \frac{exp\left(\frac{x}{2k_B T}\right)}{1+exp\left(\frac{x-W}{k_B T}\right)} \tag{S3}$$

To calculate $Z_{B\to S}(x)$ and $Z_{S\to B}(x)$, the measured values of $W = F_{tr} * \Delta X$ are averaged across opening and closing transitions using the weighted factors in Equation S3. A plot of the difference $Z = Z_{S\to B}(x) - Z_{B\to S}(x)$ with the line $Z = x$ gives the equilibrium free energy G_{BS} (shown in Figure S2 and Table S2). The G_{BS} estimates from Equation S2 and S3 are consistent with each other, suggesting that the B-to-S transitions are close to equilibrium during the stretch-release cycles.

5. Estimating the effects of bp modifications on duplex stability

The parameters obtained from the measurements were calculated at the base-pair level. First, the number of base pairs (bps) corresponding to the measured extension were estimated using an elongation per bp $x_{BS} = 0.23\ nm/bp$ as

$$n = \Delta X / x_{BS} \tag{S4}$$

The transition free energy per bp (g) was then estimated from the free energy (G_{BS}) and base pairs (n) undergoing the B-to-S transition as

$$g = G_{BS}/n \tag{S5}$$

Changes in duplex stability due to tC modifications or base rearrangements were quantified using the average transition free energy of the modified duplexes ($g_{mod} * n$) (column 6 in Table 1) minus the stability of the unmodified duplex of the same length ($g_{unmod} * n$) :

$$dG = n \cdot (g_{mod} - g_{unmod}) \quad \text{(S6)}$$

The parameters estimated are reported in Table 1.

6. B-to-S transition kinetic analysis

The B-to-S and S-to-B transitions were identified and analyzed using custom-made MATLAB codes. Firstly, the force-dependence on extension and number of transitions were calculated for a bin size of 0.5 pN by combining all the transitions (both B-to-S and S-to-B, both stretch and release cycles) from different molecules (Figure 3). Secondly, the B-to-S ($\rho(B)$) and S-to-B ($\rho(S)$) transition force histograms (with a bin size of 0.5 pN) were obtained by combing the respective transitions in stretch and release cycles from different molecules. The survival probability is defined as the probability of the dsDNA to remain in the B- or S-state at the force F. This can be computed from the B-to-S and S-to-B transition forces using:

$$P_B(F) = 1 - \frac{N(F > F_{B\to S})}{N_{B\to S}} \quad \text{and} \quad P_S(F) = 1 - \frac{N(F_{S\to B} > F)}{N_{S\to B}} \quad \text{(S7)}$$

where $N_{B\to S}$ and $N_{S\to B}$ are the total number of B-to-S transitions and S-to-B transitions over the stretch-and-release cycle, and $N(F > F_{B\to S})$ and $N(F_{S\to B} > F)$ are the numbers of B-to-S transition forces less than the given force and number of S-to-B transition forces greater than the given force, respectively. The transition rates can be calculated from the survival probabilities as follows (7):

$$k_{B\to S}(F) = -r\frac{1}{P_B(F)}\frac{dP_B(F)}{dF} \quad \text{and} \quad k_{S\to B}(F) = -r\frac{1}{P_S(F)}\frac{dP_S(F)}{dF} \quad \text{(S8)}$$

Here r is the force ramp rate (which is 5 pN/s when the pulling velocity is 50 nm/s and 20 pN/s when it is 200 nm/s). The force dependence of both transition rates is presented in Figure 3C, Figure S1M-S1R and Figure S4I-S4L. As a significant number of transitions occurs in a narrow range (vicinity of F_{tr}), the kinetic data is more reliable in this force range and was fitted to the Bell-Evans model (8-10):

$$k_{B\to S}(F) = k^0_{B\to S} \cdot \exp\left[\frac{F \cdot X_{B\to TS}}{k_B T}\right] \quad \text{and} \quad k_{S\to B}(F) = k^0_{S\to B} \cdot \exp\left[\frac{-F \cdot X_{S\to TS} + G_{BS}}{k_B T}\right] \quad \text{(S9)}$$

Here $X_{B\to TS}$ and $X_{S\to TS}$ are the distances from the B- and S-states to the transition state, respectively, and their sum is the net transition length change:

$$X_{BS} = X_{B\to TS} + X_{S\to TS} \quad \text{(S10)}$$

$k^0_{B\to S}$ and $k^0_{S\to B}$ are the extrapolated zero force transition rates that are, in turn, related to their respective transition barrier height as:

$$k^0_{B\to S} = k^0_{BS} \cdot e^{-G_{B\to TS}} \quad \text{and} \quad k^0_{S\to B} = k^0_{BS} \cdot e^{-G_{S\to TS}} = k^0_{BS} \cdot e^{-G_{B\to TS}+G_{BS}} \quad \text{(S11)}$$

Here k^0_{BS} is the universal attempt rate for the system to get over the B-to-S transition barrier and $G_{B\to TS}$ and $G_{S\to TS}$ are the zero force transition barriers that are related to the previously determined equilibrium transition free energy G_{BS} as follows:

$$G_{B\to TS} - G_{S\to TS} = G_{BS} \quad \text{(S12)}$$

Only the combination of the attempt rate and probability of overcoming the transition barrier $k^0_{BS} \cdot e^{-G_{B\to TS}}$, but not both simultaneously, can be determined from these fits.

The elongation per bp reported in Table 2 was obtained from transition distances ($X_{B\to TS}$,$X_{S\to TS}$) and number of base pairs (n) in each DNA duplex undergoing the B-to-S transition as

$$x_{B\to TS} = X_{B\to TS}/n \quad \text{and} \quad x_{S\to TS} = X_{S\to TS}/n \quad \text{(S13)}$$

7. Reconstruction of the B-to-S transition free energy profile at the transition force, F_{tr}.

The total number of forward and reverse B-to-S transitions for each stretch/release cycle (*N*) is different for the seven dsDNA oligonucleotides, and depends systematically on the average number of bps, *n*, undergoing the transition (see last column in Table 2). The scaling of the net transition free energy and extension, as well as of the extensions to and from the transition barrier with *n,* suggests that the net barrier height at F_{tr} should also be proportional to *n*, i.e. that

$$G_{TS,n} = n \cdot g_{TS} \ . \quad \text{(S14)}$$

Here $G_{TS,n}$ and g_{TS} are the B-to-S transition barrier free energies for the n bp DNA duplex, and for the single bp, respectively. (Below all lower-case values, including length and free energies are per bp, while the upper-case values are for the n-bp DNA duplex.) Therefore, the transition rates at the equilibrium transition force F_{tr} are expected to decrease exponentially with the increasing number of bps undergoing the B-to-S transition n as follows:

$$k_{B\to S}(F_{tr}) = k_{S\to B}(F_{tr}) \approx k_{BS}^{0} \cdot e^{-n \cdot g_{TS}} \tag{S15}$$

We notice that N decreases gradually from $N = 65 \pm 6$ for 3tC, with n=38.5 bps to $N = 25 \pm 3$ for Unmodified, with an average number of bps undergoing the B-to-S transition n=47.2 bps (Table 2 and Figure 4D). The average transition rate in the vicinity of the F_{tr} is ~$N/\Delta t$ where Δt~4 s is the time of the stretch/release cycle for 50 nm/s. Taking into account that the total average transition barrier is proportional to the average number of bps undergoing the transition, (Equation S15), we can estimate the transition barrier free energy per bp g_{TS} at ~F_{tr} as follows:

$$g_{TS} \approx \frac{\ln\left(\frac{N_2}{N_1}\right)}{n_1 - n_2} \approx \frac{\ln\left(\frac{65}{25}\right)}{47.2-38.5} \approx 0.11 \tag{S16}$$

Here we divided the ln of the ratio of the fastest and slowest transition rates for the shortest and longest duplexes, which is the transition free energy difference between these duplexes, by their known average bp length difference. The calculated $g_{TS} \approx 0.11$ is a very low transition barrier at F_{tr} compared to the equilibrium transition free energy per bp $g \approx 3.6$, *i.e.* $\frac{g_{TS}}{g}$~$0.03 \ll 1$. The transition free energy maximum is located at ~60% duplex extension beyond B-DNA towards S-DNA extension and has a height $G_{TS} = n \cdot g_{TS}$. The dsDNA extension beyond S-DNA requires very high energies, as the elastic modulus of S-DNA known from polymeric dsDNA studies is very high, $K_{S-DNA} = 3000\,pN$. Similarly, B-DNA contour length changes below 5% are also very energetically costly as B-DNA is known to have an elastic modulus $K_{B-DNA} = 1000\,pN$ (15,16). Taken together this information yields the B-to-S transition free energy profile at F_{tr} illustrated in Figure 5A by the red dotted line (for the n-bp DNA duplex, $G_{BS}(x) = ng(x)$). The two equally stable free energy minima at B- and S-DNA are separated by a very low free energy barrier located at ~60% extension. Below the transition extension (~0.14 nm/bp) B-DNA is relatively easy to stretch, and beyond all bps snap into the S-DNA state.

8. Reconstruction of the B-to-S transition free energy profile in the absence of DNA stretching force.

Knowing the B-to-S transition free energy profile at the transition force allows us to reconstruct the free energy profile at zero force, $g(F = 0, x)$, as follows:

$$g(F = 0, x) = g(F_{tr}, x) + \frac{F_{tr} \cdot x}{k_B T} \quad \text{(S17)}$$

Requirement of the low transition barrier positioned at 60% extension from B- towards S-DNA extension, and the existence of two equal free energy minima for the B- and S-states at F_{tr} leads to the profile shown by the dotted line in Figure 5A. For the free energy barrier to be so low at F_{tr}, the free energy at $F = 0$, $g(F = 0, x)$ should be a growing function of x at all extensions between B- and S-DNA with a slope $dg/dx \sim F_{tr}$, such that $g(F = 0, x) - \frac{F_{tr} \cdot x}{k_B T} < 0.11$ in the entire range of extensions from B- to S-DNA i.e. for $x < x_{BS} = x_{B \to TS} + x_{TS \to S} = 0.23\ nm/bp$. In other words, there should be no S-DNA free energy minimum in the absence of force. However, for the S-DNA state free energy minimum to appear at F_{tr} and x_{BS}, besides the B-DNA free energy minimum at $x = 0$, the $dg(F = 0, x)/dx$ slope should decrease abruptly beyond the transition state extension, $x_{B \to TS} \approx 0.6 x_{BS}$. Only in this case will the position of the transition state for all duplexes be the same and well-defined, as suggested by the experiments. In other words, the slope $\frac{dg(F=0)}{dx}$ should be slightly larger than F_{tr} prior to transition state extension and slightly lower than F_{tr} at larger extensions (Figure 5A, red solid line). We can estimate the transition barrier per bp in the absence of force, $g_{B \to TS}$, from the estimate of the transition barrier at F_{tr}, g_{TS}, as follows:

$$g_{B \to TS} \approx g_{TS} + g \cdot \frac{x_{B \to TS}}{x_{BS}} = 0.11 + 3.56 \cdot 0.6 = 2.25 \quad \text{(S18)}$$

Here we took into account that $g_{TS} \approx g_{B \to TS} - \frac{F_{tr} x_{B \to TS}}{k_B T} \approx g_{B \to TS} - g \cdot \frac{x_{B \to TS}}{x_{BS}}$ and that $\frac{F_{tr} x_{B \to TS}}{k_B T} \approx g \cdot \frac{x_{B \to TS}}{x_{BS}}$, and the fact that the transition midpoint lies approximately at 60% between the B- and S-extension, $\frac{x_{B \to TS}}{x_{BS}} \sim 0.6$, and our estimate of the transition barrier at F_{tr}, $g_{TS} \sim 0.11$. The S-to-B transition barrier can then be found as $g_{S \to TS} = g_{B \to TS} - g \approx 2.25 - 3.56 = -1.31$. The negative sign of $g_{S \to TS}$ means that there is no positive activation barrier to overcome while transitioning from the S-state to the B-state in the absence of force (Figure 5A, solid red line). The well-defined free energy minimum at the S-DNA extension $x_{BS} = 0.23\ nm/bp$ observed at

F_{tr} is ensured by a very steep $g(x)$ increase beyond the S-DNA extension, x_{BS}, associated with the known high elastic modulus of S-DNA, i.e. the slope of $F(x)$ above the B-to-S transition force plateau, $K_S = \frac{dF(x>x_{BS})}{dx/x_{BS}} = \frac{d^2g(F=0,x>x_{BS})}{dx^2} x_{BS} \sim 3000$ pN (17). On the other hand, B-DNA is also known to have a comparably high elastic modulus $K_B \sim 1000$ pN at the extensions exceeding its contour length by just a few percent, i.e. for $x < 0.05$ (*i.e.* up to the extension when the force reaches ~50 pN) (15,18). This means that $g(x < 0.05) = \frac{1}{2} \cdot K_B \cdot x^2 / x_{BS}$ has a very sharp minimum right around the B-DNA contour length. Further B-DNA extension at $x > 0.05$ occurs more readily, with a slope $\frac{dg(F=0,x)}{dx} \geq F_{tr}$, and with an elastic modulus $\frac{d^2g(F=0,x)}{d(x)^2} x_{BS}$ of just a few pN. At the extension ~$0.6x_{BS}$ the slope abruptly decreases to below F_{tr} , while remaining positive, and then strongly increases again beyond x_{BS} (Figure 5A).

As the whole B-to-S transition free energy profile scales with *n*, the zero force B-to-S transition barrier for an *n* bp duplex can be found as

$$G_{B\rightarrow TS,n}(F = 0) = n \cdot g_{B\rightarrow TS}(F = 0) \quad \text{(S19)}$$

Where $g_{B\rightarrow TS}(F = 0) = 2.25$ (Equation S18), and the S-to-B barrier can be found as

$$G_{S\rightarrow TS,n} = G_{B\rightarrow TS,n} - G_{BS,n} \quad \text{(S20)}$$

Here $G_{BS,n}$ is the equilibrium transition free energy for the n bp duplex known from the experiment. The negative sign of the reverse S-to-B transition barrier means that the zero-force free energy profile for the whole *n* bp duplex does not have a minimum at the S-DNA extension. Thus, for example, the unmodified DNA duplex with an average number of bps undergoing B-to-S transition n = 47 the B-to-S transition barrier is: $G_{B\rightarrow TS,47} = 47 \cdot 2.25 = 106$, and the reverse S-to-B transition barrier is $G_{S\rightarrow TS,47} = G_{B\rightarrow TS,47} - G_{BS,47} = 106 - 168 = -62$. Similar estimates of the B-to-S and S-to-B zero force transition barriers for all duplexes are collected in Table S7.

9. Effect of salt on the B-to-S transition

Any extended form of DNA (i.e. S-DNA or melted DNA) has a lower linear charge density of its phosphate groups than B-DNA, and, therefore, has lower electrostatic free energy. This effect is minimized in 1 M NaCl when all electrostatic interactions are dampened by high salt, but

becomes significant in lower salt, which equally promotes both the B-to-S transition and the internal force-induced DNA melting (with dsDNA strands staying together at the ends and internal regions of dsDNA being strand-separated). This is because both stretched internally melted DNA and S-DNA have similar extensions and are therefore equally promoted by lower salt. Fraying is energetically more favorable than internal dsDNA melting even in 1 M NaCl, due to only one DNA strand staying under the tension, while the other one is relaxed. This is why even in our 1 M NaCl experiments we observe a significant number of less stable bps fraying at forces lower than the average force of the B-to-S transition (or melting) in the rest of the molecule. Lower salt further favors the frayed DNA state, thereby promoting more fraying.

Another effect of the lower salt is that there is a higher probability of the whole duplex melting completely, instead of partially fraying and then undergoing the B-to-S transition in the rest of DNA, especially for the 3tC duplex. The reason for this observation is the following. The rest of the DNA duplex that is not fraying has a choice of going through the B-to-S transition or further melting leading to complete strand separation. The stability of the n-bp DNA duplex with respect to melting is $G_{BM}(n) = n \cdot g_{BM} - G_b$, where $G_{BM}(n)$ is the total free energy of melting of the *n* bp oligo, g_{BM} is the per bp free energy of melting, that is slightly decreasing in lower salt, G_b is the destabilizing boundary free energy that strongly increases in lower salt (11). The minimal length of the stable oligo, n_{min}, is then determined by the condition $G_{BM}(n) = 0$, i.e. $n_{min} = \frac{G_b}{g_{BM}}$, that is growing in lower salt with increasing G_b and decreasing g_{BM}. As the number of bps undergoing the B-to-S transition decreases in lower salt and growing transition force (for stabilizing 3tC modifications) the B-DNA oligo length reaches n_{min} for the given salt. At this point the B-DNA oligo melts, *i.e.* dissociates all at once, and no B-to-S transition occurs at all, which is more often observed in the 3tC duplexes that have fewer bps going through the B-to-S transition even in high salt.

Lowering the salt is also expected to decrease the average bp stability of B-DNA relative to S-DNA. According to the polyelectrolyte theory (12-14) the change in the transition free energy per bp

(in k_BT units) due to lowering of the solution ionic strength from $[Na]_1$ to $[Na]_2$ is given by the expression

$$g_{el} = \left(\frac{1}{\xi_B} - \frac{1}{\xi_S}\right) \cdot ln\left(\frac{[Na]_1}{[Na]_2}\right) \tag{S21}$$

Here $\xi = \frac{l_B}{h}$, where $l_B = 0.7\, nm$ is the Bjerrum length and h is the length per unit charge of the given state of the charged polymer. This expression works for highly charged polymers, such as B-DNA, S-DNA, ssDNA and the overstretched melted forms of DNA. The above expression can also be written and estimated for the B-to-S transition free energy change upon going from 1 M NaCl to 150 mM (0.15 M) NaCl as follows:

$$g_{el} = \frac{h_B - h_S}{l_B} \cdot ln\left(\frac{[Na]_1}{[Na]_2}\right) = \frac{0.23nm}{2 \cdot 0.7nm} \cdot ln\left(\frac{1M}{0.15M}\right) = -0.31 \tag{S22}$$

In other words, lowering the salt from 1 M to 150 mM NaCl is expected to lower the per bp B-to-S transition free energy by 0.31 k_BT. The corresponding expected B-to-S equilibrium transition force decrease is $\delta F_{tr,el} = \frac{g_{el}}{x} = \frac{-0.31 k_BT \cdot 4pN \cdot nm/k_BT}{0.23nm} = -5.42\, pN$. In reality, lowering the salt does indeed decrease the transition free energy, but only by 0.06 k_BT per bp (see Table 1), and the transition force decreases by ~1 pN, i.e. not as strongly as predicted by the polyelectrolyte theory. The explanation is, most likely, that the more extensive fraying in lower salt leaves the remaining B-DNA shorter and more GC-rich, leading to a higher per bp free energy of both melting and B-to-S transition, that partially compensates for the salt-induced destabilization. In any case, both the per bp transition free energy and force do decrease in lower salt, in qualitative agreement with the polyelectrolyte theory.

Finally, we can also learn something about the salt effect on the B-to-S transition free energy barrier. Most importantly, the Evans type analysis suggests that the extensions both from B to TS and from TS to S still scale with the number of bps undergoing the transition independently estimated from the average total measured transition extension. Also, in lower salt the transition state is still at ~60% extension from the B-state towards the S-state (see Table 2). Interestingly, lower salt results in an increase in the total number of B-to-S transitions over the stretch/release cycle for each DNA duplex, consistent with fewer bps undergoing this transition in lower salt. Moreover, just as in high salt, at 150 mM NaCl we observe more transitions occuring on average over the stretch/release cycle for 3tC modified DNA (*N=74*) compared to Unmodified (*N=36*), reflecting a larger number of bps undergoing the B-to-S transition in Unmodified (n=44.2) relative to 3tC (n=33.6). Applying the same logics as we used for

estimating the per bp transition barrier at 1 M NaCl, we can estimate the per bp transition barrier in 150 mM NaCl:

$$g_{TS}(F_{tr}, 150\ mM\ NaCl) \approx \frac{\ln\left(\frac{N_2}{N_1}\right)}{n_1 - n_2} \approx = \frac{\ln\left(\frac{74}{36}\right)}{44.2 - 33.6} = 0.07 \qquad \text{(S23)}$$

This is measurably smaller than the analogous quantity in high salt $g_{TS}(F_{tr}, 1\ M\ NaCl) = 0.11$. This is an expected result, considering that lower salt decreases all dsDNA deformation free energies, including equilibrium transition free energy. For the duplex with tens of bps this amounts to a significant difference in the transition free energy due to lower the salt. For example, for the 40-bps duplex decrease in its net transition barrier by $40\ bps \cdot (0.11 - 0.07) = 40 \cdot 0.04 = 1.6$. This, in turns, means that the B-to-S transition in the 40-bps duplex upon lowering the salt is expected to become $e^{1.6} \sim 5$ – fold faster. In other words, the kinetics of the B-to-S transition that we measure in ~40 bps duplex at ~1 M NaCl is expected to be no more than 5-fold slower than in the same length duplex at 150 mM NaCl. We conclude that the effect of salt on the B-to-S transition kinetics is rather modest.

Supplementary Figures

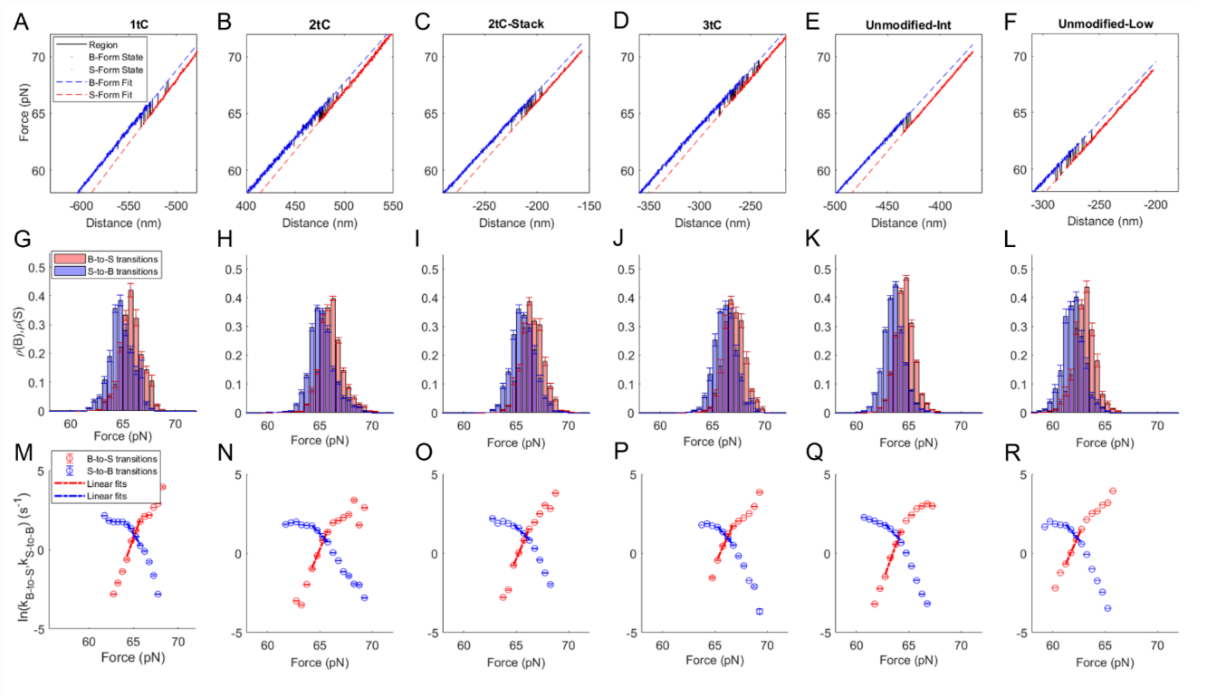

Figure S1: Effect of duplex modifications on the B-to-S transition. (A-F) A representative pull FDCs showing both B-to-S and S-to-B transitions . (G-L) Probability distribution of the transition forces of the B-to-S (red) and S-to-B (blue) transition. (M-R) B-to-S and S-to-B transition rates as a function of force for $F \sim F_{tr}$. Dotted lines are the fits of the rates vs force to the Bell-Evans model described by Equation S9. All data was obtained in 1M NaCl at 50 nm/s pulling rate.

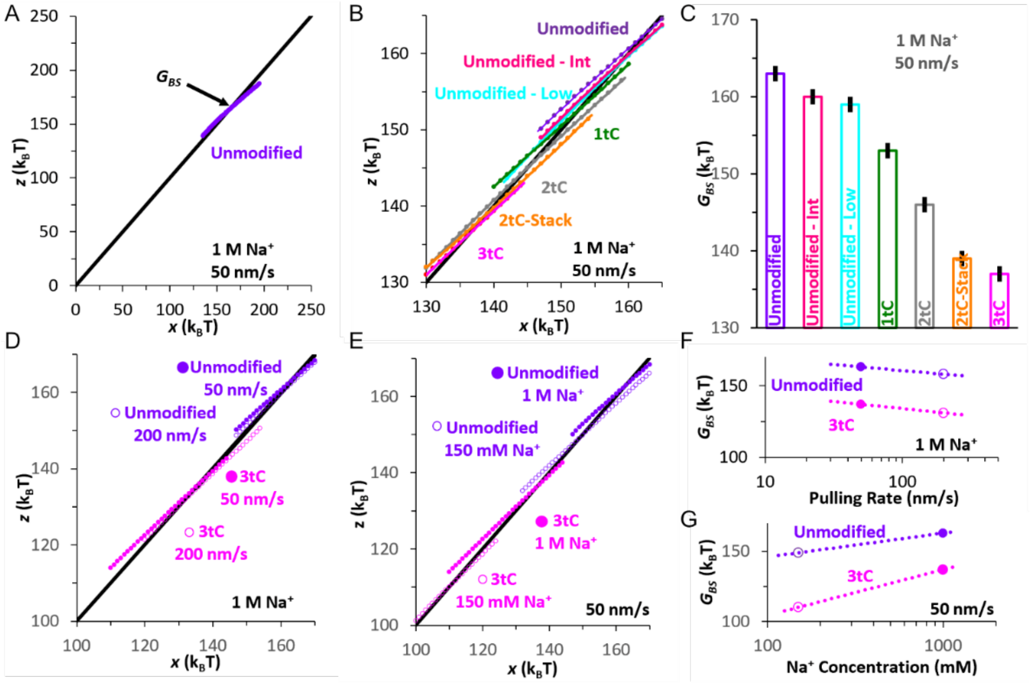

Figure S2: (A) Equilibrium transition free energy (G_{BS}) measured using Bennett's method as described in supplementary section 4 for the Unmodified duplex. According to Equation S3 the line x = z (black) crosses the transformed (non-equilibrium) transition data (violet) at the equilibrium free energy (arrow). (B) Close up and comparisons for the crossing points and equilibrium free energies for: Unmodified (violet), Unmodified-Int (pink), Unmodified-Low (cyan), 1tC (olive), 2tC (grey), 2tC-Stack (orange) and 3tC (magenta). (C) Comparisons of measured values at 1 M NaCl and a pulling rate of 50 nm/s with the same color scheme. Data for unmodified and 3tC duplexes under (D) varying pulling rates and (E) solution conditions shown in empty and full circles and summarized in (F) and (G). Measured free energy values for all duplexes are reported in Table S2.

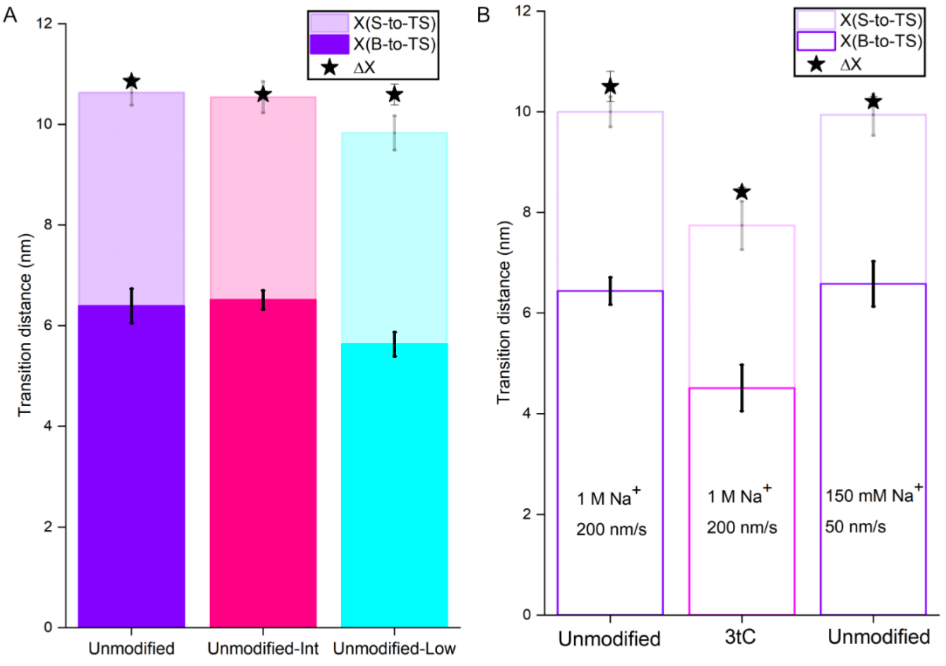

Figure S3: Estimating the distance to the transition barrier using the Bell-Evans model. Extensions ($X_{B\to TS}$) (filled-different colors) and ($X_{S\to TS}$) (transparent-different colors) from the B- and S-states, respectively, to the transition state TS. (A) Base rearranged DNA oligonucleotides: Unmodified (violet), Unmodified-Int (pink) and Unmodified-Low (cyan) duplexes. (B) Unmodified (violet-empty) and 3tC (magenta-empty) oligonucleotides at different pulling rates and salt conditions. Also presented is the sum $X_{BS} = X_{B\to TS} + X_{S\to TS}$ (net height of each bar) compared to the directly measured transition elongation ΔX (from Figure 2B), indicated with a star for each DNA duplex.

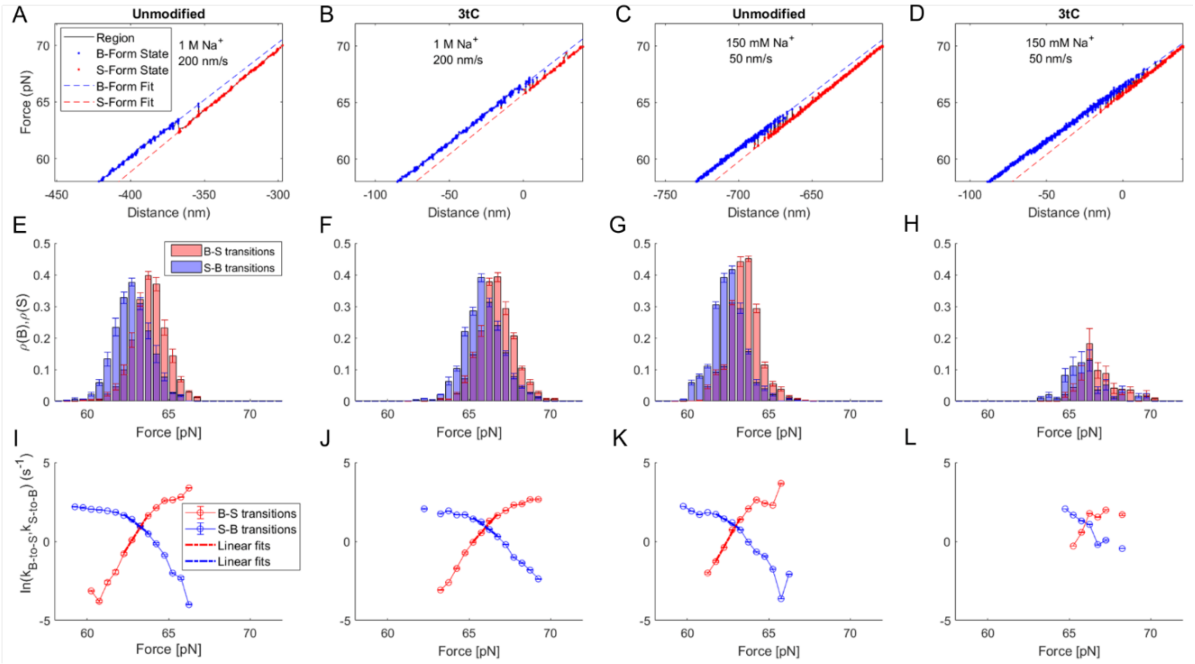

Figure S4: Effect of ionic strength and pulling rate on the B-to-S transition. Data in the first two columns of panels were obtained in 1M NaCl at 200 nm/s pulling rate. Data in the last two columns was obtained in 150 mM NaCl at 50 nm/s pulling rate. (A-D) FDCs for the studied duplexes showing both B-to-S and S-to-B transitions. (E-H) Probability distribution of the forces of the B-to-S (red) or S-to-B (blue) transition. (I-L) B-to-S and S-to-B transition rates as a function of force fitted for $F \sim F_{tr}$ with Bell-Evans model ((Equation S9) - dotted lines).

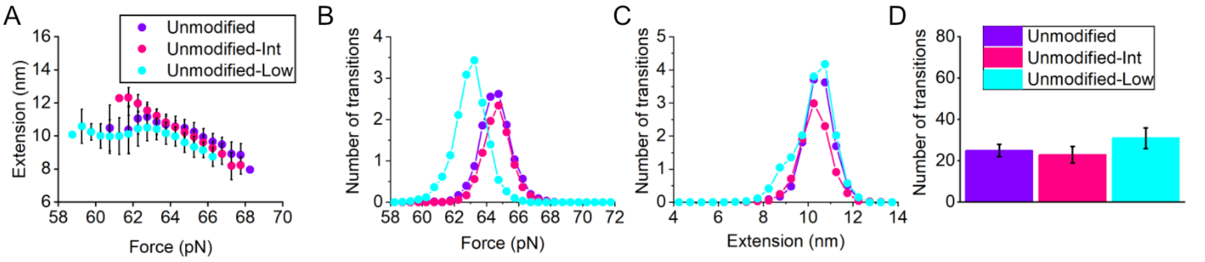

Figure S5: B-to-S transitions in dsDNA duplexes with progressively lower purine stacking interactions. (A) dsDNA oligo extension during B-to-S and S-to-B transitions vs force. (B) Average number of transitions during each stretch-release cycle as a function of the transition force and (C) extension for the Unmodified (violet), Unmodified-Int (pink) and Unmodified-Low (cyan). (D) Average number of transitions in an FDC: Unmodified (violet), Unmodified-Int (pink) and Unmodified-Low (cyan).

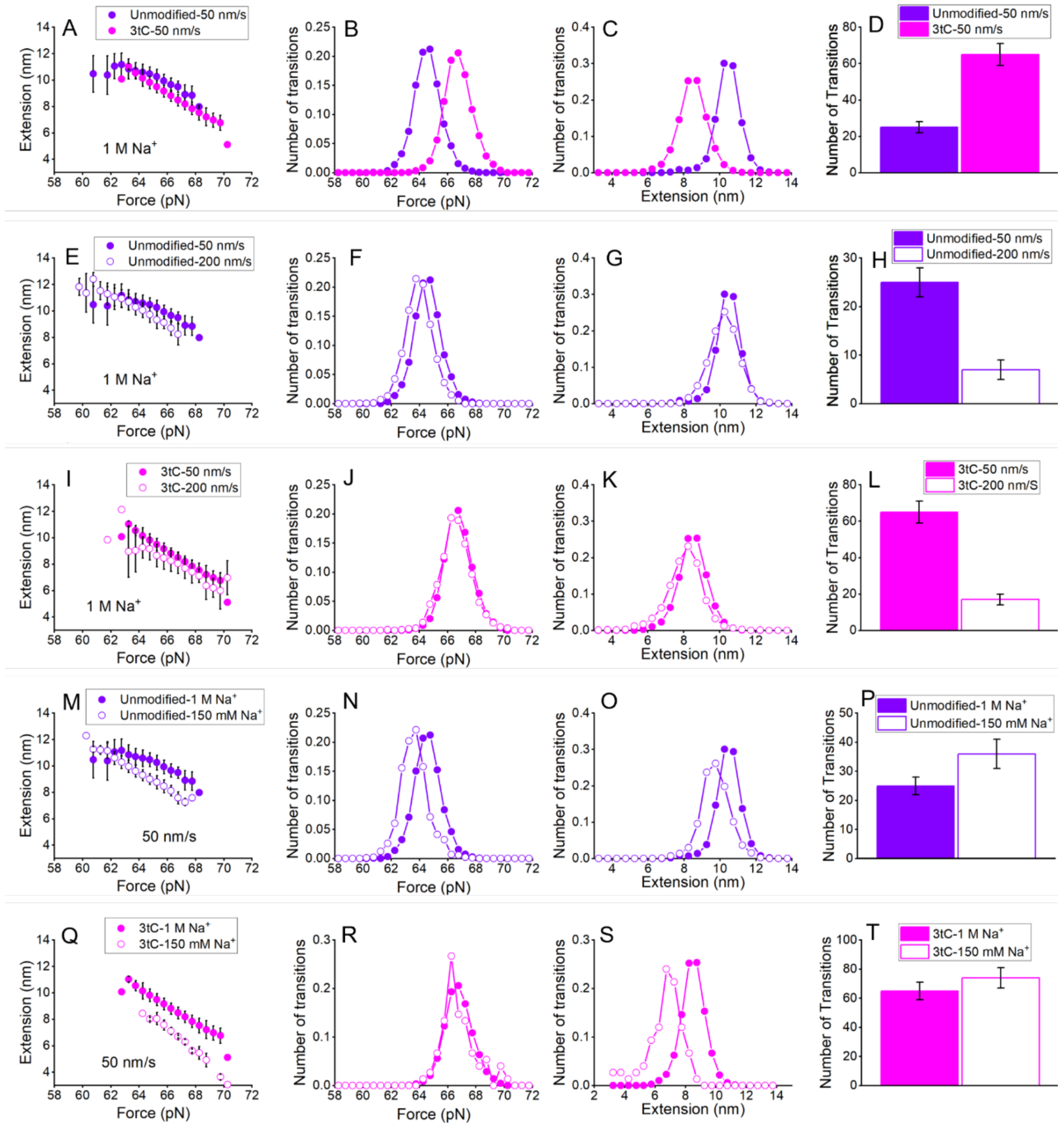

Figure S6: Stretched dsDNA oligonucleotides peel more at higher pulling rate and at low salt concentration. (A) dsDNA length changes during the transition as a function of transition force. (B) Average number of transitions during stretch-release cycle as a function of transition force normalized with total transitions. (C) Number of transitions normalized with total transitions as a function of extension. (D) Average number of transitions per stretch/release cycle for different oligonucleotides. Unmodified (50 nm/s, 1M Na^+) (violet, full), Unmodified (200 nm/s or 150 mM Na^+) (violet, empty), 3tC (50 nm/s, 1 M Na^+) (magenta, full), 3tC (200 nm/s or 150 mM Na^+) (magenta, open). Note: Very few complete pull curves were obtained for this condition as the duplex most often dissociates, making it difficult to acquire data.

Supplementary Tables

Table S1: DNA oligonucleotides used for the formation of DNA duplexes. The DNA duplexes are formed by hybridization of single stranded oligos 1 and 2 as indicated in the table. The tC modifications in oligo 1 are shown in bold red.

Oligonucleotides	Sequence
Oligo 1-1	5 -ATCAACAGAGCCACTTTGGCCCGCTGGTCGCCGCTATCGACAGGCTCAATGCTGGAGGGT-3′
Oligo 1-2	5 -ATCAACAGAGCCACTTTGGCCCGCTGGTCG**tC**CGCTATCGACAGGCTCAATGCTGGAGGGT-3′
Oligo 1-3	5 -ATCAACAGAGCCACTTTGG**tC**CCGCTGGTCG**tC**CGCTATCGACAGGCTCAATGCTGGAGGGT-3′
Oligo 1-4	5 -ATCAACAGAGCCACTTTGGCCCGCTGGTCG**tCtC**GCTATCGACAGGCTCAATGCTGGAGGGT-3′
Oligo 1-5	5 -ATCAACAGAGCCACTTTGG**tC**CCGCTGGTCG**tC**CGCTATCGA**tC**AGGCTCAATGCTGGAGGGT-3′
Oligo 1-6	5 - ATCAACAGAGCCACTATGGCGCGCTGGTCGCCGCAATCGACACGCTCAATGCTGGAGGGT-3′
Oligo 1-7	5 - ATCAACAGAGCCACTATGCGCGCGTGCACGCGCGTATGCACACGCTCAATGCTGGAGGGT-3′
Oligo 2-1	5′-ACCCTCCAGCATTGAGCCTGTCGATAGCGGCGACCAGCGGGCCAAAGTGGCTCTGTTGAT-3′
Oligo 2-2	5′- ACCCTCCAGCATTGAGCGTGTCGATTGCGGCGACCAGCGCGCCATAGTGGCTCTGTTGAT-3′
Oligo 2-3	5′-ACCCTCCAGCATTGAGCGTGTGCATACGCGCGTGCACGCGCGCATAGTGGCTCTGTTGAT-3′
Different dsDNA duplexes were formed by hybridization of oligo-1 and oligo 2	
Unmodified	Oligo 1-1 & Oligo 2-1
1tC	Oligo 1-2 & Oligo 2-1
2tC	Oligo 1-3 & Oligo 2-1
2tC-stack	Oligo 1-4 & Oligo 2-1
3tC	Oligo 1-5 & Oligo 2-1
Unmodified-Int	Oligo 1-6 & Oligo 2-2
Unmodified-Low	Oligo 1-7 & Oligo 2-3

Table S2: Comparison of the B-to-S equilibrium free energies (G_{BS}) obtained using two complementary methods as described in supplementary text.

dsDNA	G_{BS} [a] (k_BT)	G_{BS} [b] (k_BT)
Unmodified	168.0±1.7	163±1
1tC	157.9±2.1	153±1
2tC	149.5±1.3	146±1
2tC-Stack	147.2±1.9	139±1
3tC	142.7±2.0	137±1
Unmodified-Int	163.9±1.0	160±1
Unmodified-Low	160.1±3.4	159±1
Unmodified-200 nm/s	160.1±4.3	158±1
3tC-200 nm/s	134.4±1.1	131±1
Unmodified-150 mM Na^+	154.8±1.1	149±1
3tC-150 mM Na^+	123.5±5.3	110±1

[a]Calculated from transition force and extension, Equation S2. [b]Calculated using Bennett's method, Equation S3.

Table S3. Comparison of the effects of DNA duplex oligo sequence variation on duplex stability with respect to thermal melting and its B-to-S transition.

dsDNA	T_m [a] (°C)	ΔG_M [a] (k_BT)	$\Delta\Delta G_M$ [b] (k_BT)	dG [c] (k_BT)
Unmodified	96.6	235.6	0	0
Unmodified-Int	96.5	239.3	3.7	-0.01±0.65
Unmodified-Low	96.8	242.5	6.9	-3.69±0.66

[a]Calculated melting temperature, T_m and free energy, ΔG_M for dsDNA oligos with DINAMelt. [b]Calculated difference in thermal melting free energy $\Delta\Delta G_M$ of modified duplexes as compared to Unmodified. [c]Calculated free energy change dG of the B-to-S transition due to the modifications described in Table 1.

Table S4: Transition rate at transition force. The transition rate and transition force are estimated from the crossover of $\ln k_{B\rightarrow S}(F)$ and $\ln k_{S\rightarrow B}(F)$ as seen from Figure 3C, Figure S1M-S1R and Figure S4I-S4L.

dsDNA	F_{tr} (pN)	$k(F_{tr})$ (s^{-1})
Unmodified	63.94	1.03
1tC	65.18	0.98
2tC	65.53	0.99
2tC-Stack	66.01	1.12
3-tC	66.20	1.04
Unmodified-Int	64.03	0.96
Unmodified-Low	62.48	1.00
Unmodified-200 nm/s	63.34	2.31
3tC-200 nm/s	66.05	2.34
Unmodified-150 mM Na^{+}	63.09	0.97

Table S5: Extrapolated zero-force B-to-S transition rates $k^0_{B\rightarrow S}$ and $k^0_{S\rightarrow B}$ obtained by fitting experimental rates vs force dependencies (Figure 3C, Figure S1M-S1R and Figure S4I-S4L) to the Bell-Evans expressions (Equation S9).

dsDNA	$k^0_{B\rightarrow S}$ (s^{-1})	$k^0_{S\rightarrow B}$ (s^{-1})
Unmodified	$e^{-98.4}$	$e^{66.9}$
1tC	$e^{-95.9}$	$e^{65.3}$
2tC	$e^{-94.6}$	$e^{58.6}$
2tC-Stack	$e^{-84.8}$	$e^{53.3}$
3tC	$e^{-90.0}$	$e^{58.2}$
Unmodified-Int	$e^{-100.4}$	$e^{63.6}$
Unmodified-Low	$e^{-84.5}$	$e^{64.6}$
Unmodified-200 nm/s	$e^{-96.9}$	$e^{57.0}$
3tC-200 nm/s	$e^{-70.2}$	$e^{54.2}$
Unmodified-150 mM Na^{+}	$e^{-99.9}$	$e^{52.4}$

Table S6 Equilibrium properties of B-to-S transition in the wild type and 3tC modified dsDNA duplex at low salt concentration.

dsDNA	m^a	F_{tr}[b] (pN)	ΔX[b] (nm)	G_{BS}[b] (k_BT)	n^c (bps)	g^c (k_BT/bp)	dG^d (k_BT)	N
Unmodified-150 mM Na^+	55	62.7±0.1	10.2±0.1	154.8±1.1	44.2±0.3	3.50±0.01	0	36±5
3tC-150 mM Na^+	2	65.8±0.1	7.7±0.3	123.5±5.3	33.6±1.4	3.68±0.01	6.05±0.54	74±7

[a]Number of stretch and release cycles (m) obtained for 1 to 5 different bead pairs. [b]Experimental parameters from the curves. [c]Number of bps estimated using $n = \Delta X / x_{BS}$, where $x_{BS} = 0.23 \frac{nm}{bp}$ is the per bp DNA extension during the B-to-S transition known from polymeric dsDNA overstretching studies, and transition free energy per bp using $g = G_{BS}/n$. [d]Net duplex stabilization dG due to modifications was calculated using Equation S6. The reported values are the mean±standard error of mean (SEM). Measured in 10 mM Tris pH 7.4, 1 mM EDTA, 1 M NaCl, at 23±1°C. Unmodified and 3tC duplexes were measured at 150 mM NaCl and 50 nm/s pulling velocity. The data for 3tC at 150 mM NaCl is shown here and compared with Unmodified due to insufficient number of curves taken. Note: The upper-case values including length and free energies are for the n-bps DNA duplex while all lower-case values, are per bp. Also, G with subscripts denotes an equilibrium free energy, while the use of an arrow in the subscripts refers to the free energies of the transition state relative to the B- or S-states, respectively.

Table S7. Reconstruction of the B-to-S free energy profile at $F = 0$ from the DNA oligonucleotide length dependence of transition kinetics.

dsDNA	n^a (bps)	F_{tr}[a] (pN)	N[a]	$G_{BS,n}$[a] (k_BT)	$G_{B\rightarrow TS,n}$[b] (k_BT)	$G_{S\rightarrow TS,n}$[b] (k_BT)
Unmodified	47.2±0.4	63.68±0.12	25±3	168.0±1.7	106.0±6.7	-61.8±6.9
1tC	43.5±0.6	64.89±0.14	28±3	157.9±2.1	97.9±6.2	-60.0±6.6
2tC	41.0±0.4	65.22±0.14	42±4	149.5±1.3	92.2±5.8	-57.2±5.9
2tC-Stack	40.1±0.5	65.68±0.19	44±5	147.2±1.9	90.2±5.7	-57.0±6.0
3-tC	38.5±0.6	66.22±0.18	65±6	142.7±2.0	86.6±5.6	-56.1±5.9
Unmodified-Int	46.0±0.3	63.67±0.07	23±4	163.9±1.0	103.5±6.5	-60.4±6.5
Unmodified-Low	46.1±0.9	62.18±0.20	31±5	160.1±3.4	103.7±6.8	-56.4±7.6

[a]Parameters obtained from the experiments and already reported in Table 1 and 2. [b]$G_{B\rightarrow TS,n}$ was obtained by multiplying the zero-force barrier per bp g_{B-TS} by the number of bps in the transition n Equation S19 and $G_{S\rightarrow TS,n}$ using Equation S20. The reported values are the mean± SEM. The standard errors for [b] were estimated using propagation of errors.

Supplementary References

1. Bosaeus, N., El-Sagheer, A.H., Brown, T., Akerman, B. and Norden, B. (2014) Force-induced melting of DNA--evidence for peeling and internal melting from force spectra on short synthetic duplex sequences. *Nucleic Acids Res*, **42**, 8083-8091.
2. Bosaeus, N., El-Sagheer, A.H., Brown, T., Smith, S.B., Akerman, B., Bustamante, C. and Norden, B. (2012) Tension induces a base-paired overstretched DNA conformation. *Proc Natl Acad Sci U S A*, **109**, 15179-15184.
3. Smith, S.B., Cui, Y. and Bustamante, C. (2003) Optical-trap force transducer that operates by direct measurement of light momentum. *Methods Enzymol*, **361**, 134-162.
4. Severino, A., Monge, A.M., Rissone, P. and Ritort, F. (2019) Efficient methods for determining folding free energies in single-molecule pulling experiments. *Journal of Statistical Mechanics: Theory and Experiment*, **2019**.
5. McCauley, M.J., Rouzina, I., Manthei, K.A., Gorelick, R.J., Musier-Forsyth, K. and Williams, M.C. (2015) Targeted binding of nucleocapsid protein transforms the folding landscape of HIV-1 TAR RNA. *Proc Natl Acad Sci U S A*, **112**, 13555-13560.
6. Collin, D., Ritort, F., Jarzynski, C., Smith, S.B., Tinoco, I., Jr. and Bustamante, C. (2005) Verification of the Crooks fluctuation theorem and recovery of RNA folding free energies. *Nature*, **437**, 231-234.
7. Dudko, O.K., Hummer, G. and Szabo, A. (2006) Intrinsic rates and activation free energies from single-molecule pulling experiments. *Phys Rev Lett*, **96**, 108101.
8. Evans, E. and Ritchie, K. (1997) Dynamic strength of molecular adhesion bonds. *Biophys J*, **72**, 1541-1555.
9. Evans, E. (2001) Probing the relation between force - Lifetime - and chemistry in single molecular bonds. *Annu Rev Bioph Biom*, **30**, 105-128.
10. Landuzzi, F., Viader-Godoy, X., Cleri, F., Pastor, I. and Ritort, F. (2020) Detection of single DNA mismatches by force spectroscopy in short DNA hairpins. *J Chem Phys*, **152**, 074204.
11. Bloomfield, V.A., Crothers, D.M. and Tinoco, I. (2000) *Nucleic Acids: Structure, Properties, and Functions*. University Science Books.
12. Frankkamenetskii, M.D., Anshelevich, V.V. and Lukashin, A.V. (1987) Polyelectrolyte Model of DNA. *Usp Fiz Nauk+*, **151**, 595-618.
13. Bond, J.P., Anderson, C.F. and Record, M.T., Jr. (1994) Conformational transitions of duplex and triplex nucleic acid helices: thermodynamic analysis of effects of salt concentration on stability using preferential interaction coefficients. *Biophys J*, **67**, 825-836.
14. Wenner, J.R., Williams, M.C., Rouzina, I. and Bloomfield, V.A. (2002) Salt dependence of the elasticity and overstretching transition of single DNA molecules. *Biophys J*, **82**, 3160-3169.
15. Smith, S.B., Cui, Y.J. and Bustamante, C. (1996) Overstretching B-DNA: The elastic response of individual double-stranded and single-stranded DNA molecules. *Science*, **271**, 795-799.
16. Bianco, P., Bongini, L., Melli, L., Dolfi, M. and Lombardi, V. (2011) PicoNewton-millisecond force steps reveal the transition kinetics and mechanism of the double-stranded DNA elongation. *Biophys J*, **101**, 866-874.
17. Zhang, X., Chen, H., Le, S., Rouzina, I., Doyle, P.S. and Yan, J. (2013) Revealing the competition between peeled ssDNA, melting bubbles, and S-DNA during DNA overstretching by single-molecule calorimetry. *Proc Natl Acad Sci U S A*, **110**, 3865-3870.
18. Bongini, L., Melli, L., Lombardi, V. and Bianco, P. (2014) Transient kinetics measured with force steps discriminate between double-stranded DNA elongation and melting and define the reaction energetics. *Nucleic Acids Res*, **42**, 3436-3449.
